# Greater Celandine's Ups and Downs−21 Centuries of Medicinal Uses of *Chelidonium majus* From the Viewpoint of Today's Pharmacology

**DOI:** 10.3389/fphar.2018.00299

**Published:** 2018-04-11

**Authors:** Sylwia Zielińska, Anna Jezierska-Domaradzka, Magdalena Wójciak-Kosior, Ireneusz Sowa, Adam Junka, Adam M. Matkowski

**Affiliations:** ^1^Pharmaceutical Biology and Botany, Wrocław Medical University, Wrocław, Poland; ^2^Botanical Garden of Medicinal Plants, Wrocław Medical University, Wrocław, Poland; ^3^Analytical Chemistry, Medical University of Lublin, Lublin, Poland; ^4^Pharmaceutical Microbiology and Parasitology, Wrocław Medical University, Wrocław, Poland

**Keywords:** isoquinoline alkaloids, chelidonine, chelerythrine, cytotoxic, anti-inflammatory, anti-microbial

## Abstract

As antique as Dioscorides era are the first records on using Chelidonium as a remedy to several sicknesses. Inspired by the “*signatura rerum*” principle and an apparent ancient folk tradition, various indications were given, such as anti-jaundice and cholagogue, pain-relieving, and quite often mentioned—ophthalmological problems. Central and Eastern European folk medicine has always been using this herb extensively. In this region, the plant is known under many unique vernacular names, especially in Slavonic languages, associated or not with old Greek relation to “chelidon”—the swallow. Typically for Papaveroidae subfamily, yellow-colored latex is produced in abundance and leaks intensely upon injury. Major pharmacologically relevant components, most of which were first isolated over a century ago, are isoquinoline alkaloids—berberine, chelerythrine, chelidonine, coptisine, sanguinarine. Modern pharmacology took interest in this herb but it has not ended up in gaining an officially approved and evidence-based herbal medicine status. On the contrary, the number of relevant studies and publications tended to drop. Recently, some controversial reports and sometimes insufficiently proven studies appeared, suggesting anticancer properties. Anticancer potential was in line with anecdotical knowledge spread in East European countries, however, in the absence of directly-acting cytostatic compounds, some other mechanisms might be involved. Other properties that could boost the interest in this herb are antimicrobial and antiviral activities. Being a common synanthropic weed or ruderal plant, *C. majus* spreads in all temperate Eurasia and acclimates well to North America. Little is known about the natural variation of bioactive metabolites, including several aforementioned isoquinoline alkaloids. In this review, we put together older and recent literature data on phytochemistry, pharmacology, and clinical studies on *C. majus* aiming at a critical evaluation of state-of-the-art from the viewpoint of historical and folk indications. The controversies around this herb, the safety and drug quality issues and a prospective role in phytotherapy are discussed as well.

## Introduction

“No less extraordinary is the property of the herb Celandine; which, it is said, if any man shall have this herb, with the heart of a Mole, he shall overcome all his enemies, all matters in suit, and shall put away all debate,” and “if before named herb be put upon the head of a sick man, if he shall die, he shall sing anon with a loud voice, if not, he shall weep”; and “it bringeth the business begun to an end,” so wrote Albertus Magnus in thirteenth century A.D. (Best and Brightman, 1999). Nowadays, mankind would definitely benefit from such a miraculous remedy. Unfortunately, such claims about *Chelidonium majus* L.—the Greater Celandine, have not been verified according to the modern evidence-based approach (but no data on rigorous testing toward such properties actually exist in the literature). However, through years of investigations, many other properties ascribed to this inconspicuous but characteristic plant have been confirmed or re-discovered. Several others could not be positively confirmed. Despite the widespread use in folk medicine and in official phytotherapy, both in Europe and in Traditional Chinese Medicine, the celandine herb did not join the most popular herbal remedies such as chamomile, valerian, St. John's wort or ginseng. It has been listed in pharmacopeias and sold in pharmacies in parallel to the spontaneous collection by people seeking drugs against gastrointestinal disorders, cancer, infections, but especially against warts and any skin protuberances. This was the reason to combine the available historical and ethnobotanical data with the state-of-the art in pharmacology of *C. majus* and its components in the present review (Supplementary Figure 1). To date, only a couple of papers have provided review of pharmacological and phytochemical knowledge with the EMA assessment report from 2011 (European Medicines Agency, 2011) and the review (Gilca et al., 2010), being the most recent and comprehensive ones. Biswas (2013) has published a short review with overview of selected bioactivities but only covering a fraction of available literature and suggesting future directions of research. Similar approach to review the *C. majus* properties was used by Arora and Sharma (2013) who summarized some activities based on selected literature and included pharmacognostic characteristics. Gilca et al. (2010) has classified the pharmacological evidence into the categories related to traditional usage, also from the viewpoint of the TCM, such as anti-infectious, spasmolytic, gastric and hepatic, and anticancer. They also listed traditional indications that had not been confirmed by modern research, such as diuretic, anti-edema, expectorant and antitussive, pulmonary, and ophthalmological diseases. Some current information is also available in herbal books and compendia (Wichtl, 2004) or in the Internet. The latter source is of course difficult to verify.

Therefore, we chose to include a possibly high number of available literature, by selecting records from database search (PubMed, Scopus, Google Scholar) with the term “chelidonium” or “celandine” and manually eliminating papers pertaining to field botany, ecology and other aspects not relevant to medicinal use of this plant. Some references that have been reviewed earlier (e.g., in Colombo and Bosisio, 1996) were also not cited directly if the information was redundant. Some information about the historical applications and folk medicine in Central and Eastern Europe were obtained from sources available in local libraries. In particular, we describe the phytochemical composition of various parts of the plant, the methods used for obtaining extracts and analysis of the herbal material. Further, we critically summarize the most credible research on bioactivity and clinical efficacy of various products and substances from *C. majus*. In addition, the highly debated and controversial issue of the patented, apparently semi-synthetic drug NSC-631570 (Ukrain®) promoted mainly as a cancer cure was discussed (Ernst and Schmidt, 2005). With this review, we hope to encourage more research and attract interest to this quite common but not always adequately respected weed.

## Botanical description

*C. majus* L. (*Papaveraceae*^1^) is a short-lived hemicryptophyte. It has up to 1 m high stem, branched and sparsely pubescent. The alternately placed leaves are light bluish at the bottom and green at the top. The basal leaves are long-petioled, with the obovated in contour, pinnatosected leaflets with 5–7 lobed segments. The apical leaves are short-petioled, with 3-lobed leaflet. From April to October the plant produces umbellate inflorescences with 2–6 flowers, which have 4 bright yellow petals and two whitish, early dropping sepals. The fruit is an elongated (3 cm), pod-shaped, multiseeded capsule, dehiscent with two valves. The seeds are shiny, ovate and dark brown or black, with elaiosomes. The underground part is a short tap root with numerous and elongated lateral roots. The whole plant contains yellow to orange latex. *C. majus* grows in the lowlands and foothills in leafy forests, in brushwood, parks, gardens, on the roadsides and around buildings. It prefers moist soils rich in nitrogen and organic matter (Zarzycki et al., 2002).

### Distribution area

*C. majus* is native in Europe, western and central part of Asia and in northern Africa. It occurs from Portugal in the West, to Central, Eastern to Northern Europe. The Asian range covers Turkey, Iran, Kazakhstan, Mongolia, Caucasus, and Siberia. In North America it is an introduced plant.

### Taxonomy and nomenclature

Until the mid-twentieth century the genus *Chelidonium* L. was monotypic with *C. majus* L. as the only species. In 1982 Krahulcowa based on cytotaxonomic study of *C. majus* L. *sensu lato*, proposed to divide the genus into two microspecies. She proposed *C. majus* L. (2n = 12) distributed in Europe, Siberia, and China and a new species *C. asiaticum* (Hara) Krahulcova (2n = 10), an East Asian vicariant (Krahulcová, 1982). Aside from the difference in chromosome numbers and distribution area, *C. asiaticum* slightly differs morphologically from *C. majus*. It is more hairy, with narrower and sharper leaf lobes. Within *C. majus* she distinguished European *C. majus* L. subsp. *majus* with more laciniate lobes of leaves, and *C. majus*. subsp. *grandiflorum* (DC.) Printz, in South Siberia and China (Krahulcová, 1982).

(Krahulcová, 1982) Name of the genus derives from Greek (χ*ελιδóνιoν*) with *chelidon* (χ*ελιδóν*) meaning swallow (a bird) for the plant usually blooms simultaneously with arrival of these birds. The specific epithet *majus* in Latin means bigger.

**Common English name**: Greater celandine.

The name *celandine* originates from Medieval Latin word *celidonia*, a phonetic variant of Latin *chelidonia*, which was recorded by Pliny. Similarly, the German name, *Schöllkraut*, comes from *Schellkraut* (Bauhin, 1651), which is derived from Latin-Greek *chelidonium* (Waniakowa, 2015).

#### Common and folk names in some european languages

Albanian—latrapeci, bar saraleku; Belarussian—padtynnik, barodaunik (wart herb); Bosnian—rosopas; Bulgarian—zmiysko mlako; Croatian—zmijino mlijeko, rosopas; Czech—vlaštovičník, celadona, celduně, cen dalie, dravnicovina, hadí mlíč, krkavník; Dutch—stinkende gouwe; English—Tetterwort, devils's milk, rock poppy; French—grande chélidoine Éclair, herbe aux boucs, herbe a l'hirondelle; German—Schöllkraut, Gilbkraut, Goldwurz, Schwalbenkraut, Warzenkraut; Italian—celidonia, cinerognola, Montenegrin—rusopas, rusa; Polish—glistnik jaskólcze-ziele, celidonia, cyndalia, cencylja, glistewnik, gliśnik, niebospad, złoty groszek, złotnik, zółte ziele, zółte kwiatki, roztopaść; Ukrainian—hladyshnyk, hnystnyk, zhovtyi molochay, smetannyk, chystotil; Romanian: rostopască; Russian—chistotel; Rusyn—rostopast'; Serbian—rusopas, rusa, rusomača; Spanish—Golondrinera, Hierba de las verrugas (wart herb).

## *Chelidonium majus* in folk medicine

### History of usage

*C. majus* has been known as medicinal species since the very Antiquity. Medicinal properties of *C. majus* were described by Dioscorides and Pliny the Elder in the first century AD. Dioscorides in *De Materia Medica* states that celandine begins to blossom when the swallows arrive and withers when they depart. He also refers to a lore saying that swallows restore sight to their blind nestlings with use of celandine (Osbaldeston and Wood, 2000). Pliny the Elder repeats these accounts in *Naturalis Historiae* (Jones, 1966).

The foremost medicinal use of celandine, described since the ancient times until the sixteenth century, was treating visual impairment and eye diseases. For such conditions, Dioscorides advised to use herb juice, boiled with honey in a brass vessel. The juice could also be dried in the shade and the resulting small pellets were ingredients of other medicinal products. According to Dioscurides celandine soaked in wine together with anise fruits was helpful in treating jaundice and dermatologic disorders such as herpes. Besides, chewing on a root relieved toothache (Osbaldeston and Wood, 2000). Pliny advised a kind of eye lotion, which takes its name, *chelidonia*, from the name of the plant (Jones, 1966).

Celandine was an admired medicinal plant during the Middle Ages, mostly used to cure eye diseases, for throat cleansing, treatment of ulcers and skin eczema as well as against colic and jaundice (Mayer et al., 2003). In 1080, French monk and physician Odo Magdunensis wrote *De viribus herbarum*, also known as *Macer floridus*, a botanical poem describing medical effects of 77 plants. One of the chapters of *M. floridus* deals with medicinal application of celandine in “*v*isual impairment as well as skin and liver conditions” (Mayer et al., 2003). Hildegard of Bingen wrote about celandine in her work *Liber subtilitatum diversarum naturarum creaturarum* created during 1150–1160 A.D. and finally published in 1533. Hildegard recommended celandine juice to enhance sight and juice mixed with tallow as a cure for skin ulcer (Mayer et al., 2003). Moreover, celandine would be a strong *aphrodisiacum*, but causing infertility on ladies. Breathing the smell of the plant by spouses prevents arguments. Celandine was also recommended by to treat jaundice and against hair overgrowth (Czekański, 2007).

Since the sixteenth century, according to Paracelsus's signature doctrine, celandine was used to treat jaundice and liver diseases (Rostanski, 1997). C. *majus* was described in comprehensive botanical medical works of scholars such as: Joannes Minoritanus, Marcello Vergili, Hieronymus Bock, Leonhard Fuchs, Pierandrea Matthioli, and Adam Lonicer. The authors refer to antique sources and recommend using celandine to treat eye and skin conditions. According to Lonicer, to cure various dermatologic diseases, known in those days as “leprosy,” the juice of the root of celandine had to be applied on the skin, conjointly with drinking the juice mixed with syrup of common fumitory (*Fumaria officinalis*) twice a day for 9 days (Mayer et al., 2003). At the turn of the sixteenth and seventeenth century two large works on herbs and their applications were written in Poland. The first is *Herbarz Polski* by Marcin of Urzedowo, printed in 1595 and the second is *Herbal* by Simon Syrenius, published in 1613. Marcin of Urzedowo described, based on works of Dioscorides, uses of celandine to treat eye diseases, jaundice, wounds, toothache, and colic. To cure sight impairment, herb juice boiled with honey or pellets made of juice dried in shade should be used. Cataract, on the other hand, should be treated with juice draining from a broken stem or root of the plant. In case of jaundice, root of celandine should be boiled in white wine and the decoction should be drunk for a few days. The crashed root with wine was drunk to treat colic and applied on wounds. Applying a piece of root of celandine to an aching tooth, relieved the pain (Marcin of Urzedowo, 1595).

Simon Syrenius, described celandine-based recipes used in eye diseases. The main ingredient of such medicines is fresh celandine juice. As one of the few authors, Syrenius considered the juice irritative and therefore recommended mixing it with small amounts of vinegar, milk, or rose water. He also advised drinking a decoction of celandine roots cooked in wine with anise or a mixture of powdered root with vinegar before bedtime to treat jaundice, colic, and stomach ache. In case of toothache he advised to rub teeth with powdered root with vinegar. Body ulcers and scabs on the head skin can be cured with a salve of powdered root mixed with pork fat and vinegar. Alternatively, the powdered root alone could be put directly on the ulcers. Syrenius also describes the diaphoretic and diuretic effects of the herb along with the roots or the root itself. The root boiled in wine has a diuretic effect. As diaphoretic remedy Syrenius recommended taking dry bath of celandine herb, which would cause extensive sweating and expulsion of excess water from the body as well as drinking decoction from roots boiled in rose vinegar or white wine with great water dock (*Rumex hydrolapathum*). In addition, the celandine could be used to dye the hair yellow, and to lighten the freckles and hyperpigmentation on the face (Syrenius, 1613).*The British Flora Medica* (Barton and Castle, 1845) cites the traditional applications of *C. majus* in treatment of jaundice, visceral obstructions, fevers, dropsies, scrofula, syphilitic affliction, gout, cataract, ophtalmia, and specks as described previously by Dioscorides and Galen.

In countries, where *C. majus* is the native species, it became one of the most widespread drug of folk herbal medicine. The scope of its applications in folk medicine shows high similarity among many regions of Central and Eastern Europe. It is worth to mention its prevalent application to treat warts, eczema and other skin diseases, gastrointestinal parasites, jaundice, and liver complaints, inflammatory eye infections and other diseases, including cancer.

### Skin diseases

In folk medicine, *C. majus* was most commonly used to remove warts. Herb juice or latex were used most frequently for this purpose, however, use of leaves and flowers was also noted. In Poland it was common to rub the fresh juice from the broken stem of the celandine onto warts (De Verdmon, 1936; Kuźniewski and Augustyn-Puziewicz, 1999; Kujawska et al., 2016). In the Bieszczady Mountains and in the Podkarpacie Region (S-E Poland) the juice was used directly on warts, or they were first scrubbed off and then the juice was applied on the wound (Szary, 2013). A cataplasm made of flowers that was supposed to be changed every few days was used in the Kielce Region (Central Poland) (Szot-Radziszewska, 2012). Occasionally, fresh leaves were also used (Kujawska et al., 2016). The juice of the aerial part of the plant was used in the Ukrainian Carpathians (Szary, 2010, 2013) and in Russia (Zevin et al., 1997). The herb juice was applied to treat warts also in the Balkan countries (Redžič, 2007; Tita et al., 2009; Menković et al., 2011; Mustafa et al., 2012; Koleva et al., 2015) as well as in Central Italy (Menale et al., 2006) and Great Britain (Barton and Castle, 1845). Other dermatologic conditions were also treated. All around Poland it was common to apply fresh leaves or juice on wounds. In Podolia (Ukraine) corns were treated by rubbing with a root of celandine and by application of fresh leaves. After a week, corns softened and ruptured. A salve from celandine, olive oil, fir resin and beeswax was a remedy for pustules (Kujawska et al., 2016). In the Bieszczady mountains (Polish-Slovakian-Ukrainian frontier), juice of celandine was applied to eczema and cuts, and decoction of root was used for baths and rinse for dermatologic conditions (Szary, 2013). In the Rzeszowszczyzna (S-E Poland) region leaves were applied to ulcers to stimulate picking up and rupture (Wdowiak and Bielecka-Grzela, 2013). The juice was also used to *lighten freckles* (Kuźniewski and Augustyn-Puziewicz, 1999). In Russia the juice of aerial parts was used in the treatment of skin wounds, skin irritation, allergic rashes and dermatitis, leaves, and flowers in the treatment of boils (Mamedov et al., 2004). The aerial parts of the plant were used by the people of Montenegro to cure blisters, rashes, and scabies (Menković et al., 2011). In Central Serbia, juice was applied directly on skin in skin eruptions, psoriasis and eczema (Jarić et al., 2007).

### Liver diseases

*C. majus* is one of the best-known folk medicine remedy for jaundice and liver diseases, such as inflammation, spastic conditions, and gallstones. In Poland, infusion made of young celandine leaves was used as a cholagogue and to regulate action of the digestive tract (Kuźniewski and Augustyn-Puziewicz, 1999). Jacques (De Verdmon, 1936) in case of jaundice advised infusion made of half of a teaspoon celandine per cup. All around Poland it was common to bath children with jaundice in celandine and to give celandine infusion to drink (Kujawska et al., 2016). In the Bieszczady and in the Ukrainian Carpathians, herb infusion was drunk to relieve liver conditions (Szary, 2010). In Western Ukraine, infusion was used as relaxant in colic attacks (Szot-Radziszewska, 2007). Also in Balkan countries, celandine was employed in the treatment of liver disorders. In the Albanian Alps to treat hepatitis a decoction of fresh aerial parts has been drunk with sugar in small portions—half coffee cup (Pieroni et al., 2005). In Serbia celandine was used internally for inflammation of the gallbladder, bile duct, jaundice, and hepatitis (Jarić et al., 2007; Šavikin et al., 2013). The use of celandine is similar in Gollak region in Kosovo (Mustafa et al., 2012), in the Prokletije Mountains (Menković et al., 2011) and in Zagori in Epirus, North-West Greece (Vokou et al., 1993).

### Against digestive tract parasites

Polish name of *C. majus* “glistnik” (roundworm herb) comes from a common traditional usage of this plant to expel roundworms. For this reason decoction of the herb had to be drunk for 12 days (De Verdmon, 1936). In the Bieszczady Mountains children were bathed in decoction of the celandine herb and were given celandine infusion to drink (Szary, 2013). Decoction of seeds was also used in the Kielce Region (Szot-Radziszewska, 2012). In western Ukraine, infusion of the herb was prepared (Szot-Radziszewska, 2007).

### Eye diseases

Contrary to ancient phytotherapy, celandine was rarely used to treat eye conditions in folk medicine. Wdowiak (2015) reports that in the Podolia Region (Ukraine) drops of celandine juice mixed with vodka were put into eyes. In west Ukraine a tincture made from celandine was applied. Moreover, a popular belief among people of the Podolia Region, as well as the Lubelszczyzna and Podkarpacie Regions (Eastern Poland) said that feces of a swallow can cause sight loss, if they fall into the eye, which can be only cured by *C. majus*. In the small town Giby, of Polish-Lithuanian-Belarusian borderland, the celandine pollen was used against eye infections (Kujawska et al., 2017).

### Other usage

People of S-W Romania and Zagori in Greece were applying celandine as diuretic (Vokou et al., 1993; Tita et al., 2009). In the central Serbia and in Podolia (Ukraine), celandine was considered a remedy for gout. Occasionally, it was used as tonic and stimulant of cardiac functions, also increasing blood pressure (Vokou et al., 1993). In the Bieszczady Mountains people incensed aching teeth with the smoke from the burning herb (Szary, 2015). In Poland, juice was used internally to cure hydropsy (Kujawska et al., 2016). In Romania it was esteemed as an antidote for snake venom (Tita et al., 2009). People of Russian descent, called Russlanddeutschen, living in S-W Germany used celandine as depurative (Pieroni and Gray, 2008). Among the Hutsuls living on the Ukrainian side of Bukovina (S-W Ukraine), tea from aerial parts of celandine was employed in the treatment of cancer (Sǒukand and Pieroni, 2016). In Bosnia and Herzegovina it was used to cure cancer of lungs (Redžič, 2007). In veterinary treatments, herb decoction was given to cows suffering from inflammation of the udder, in case of dermatologic conditions animals were rubbed with leaves. Furthermore, cows were given root to eat to cause vomit to relieve bloat. Besides, herb overcooked in milk was applied to ulcers (Kujawska et al., 2016). In the Bieszczady Mountains celandine was a symbol of purification of living world from threatening death, it was used as talismans to protect from demons (Szary, 2015). Dried herb was used to incense the interiors of huts to deter flies and mosquitos as well as during plague and other epidemics. Grains soaked in celandine juice were used as fish and bird poison (Szary, 2013).

## Phytochemistry

For the therapeutic purposes, dried herb of *C. majus* is used (European Pharmacopeia). In some regions (Central and Eastern Europe) roots are also exploited. European Pharmacopeia calls for total alkaloid content as chelidonine [1], assayed spectrophotometrically with additional TLC screening and microscopic authentication.

### Alkaloids

**Figure 1:**
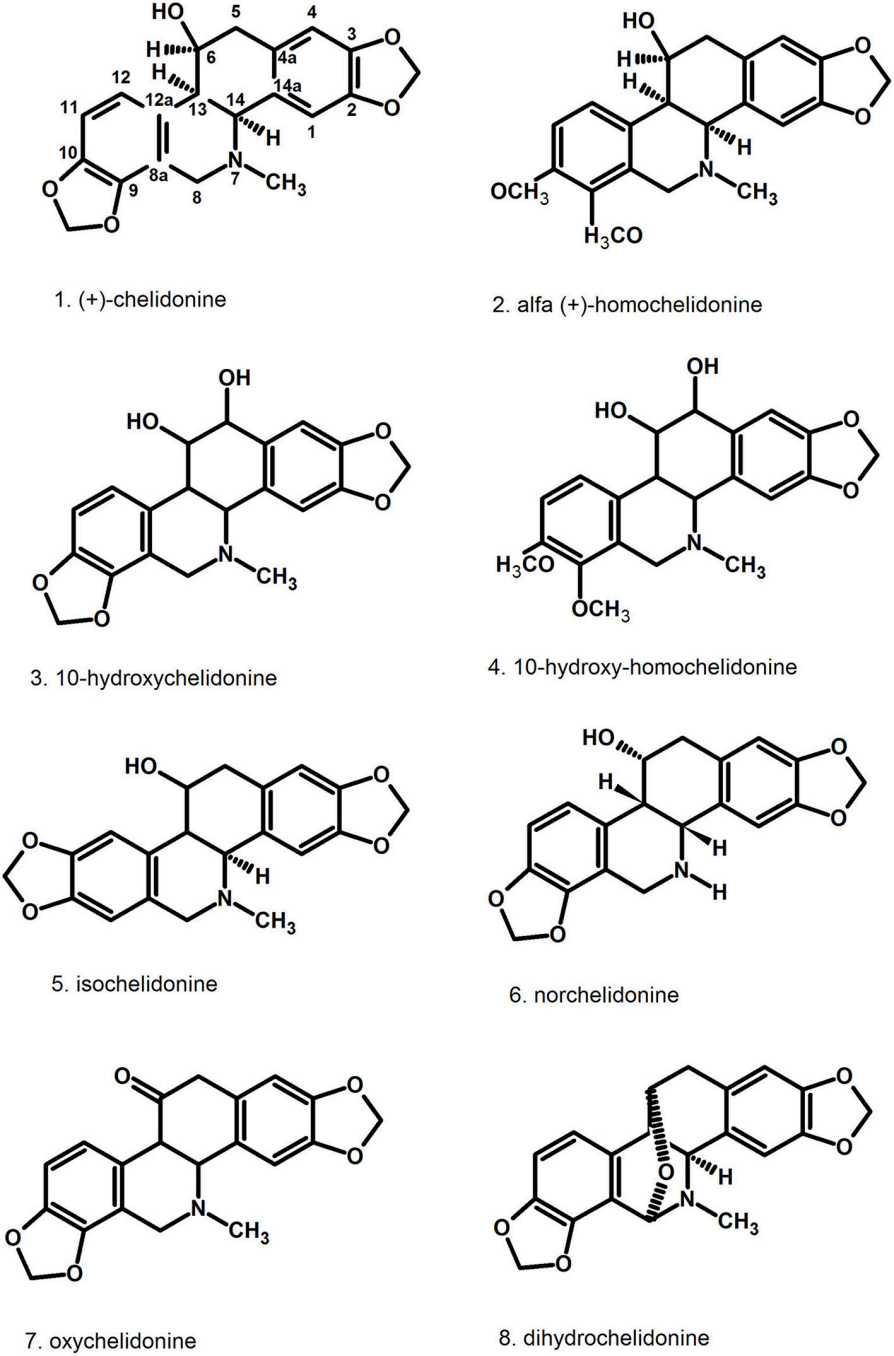
Structures of phenanthridine (3,4-benzoisoquinoline) alkaloids—chelidonine [1] derivatives.

**Figure 2:**
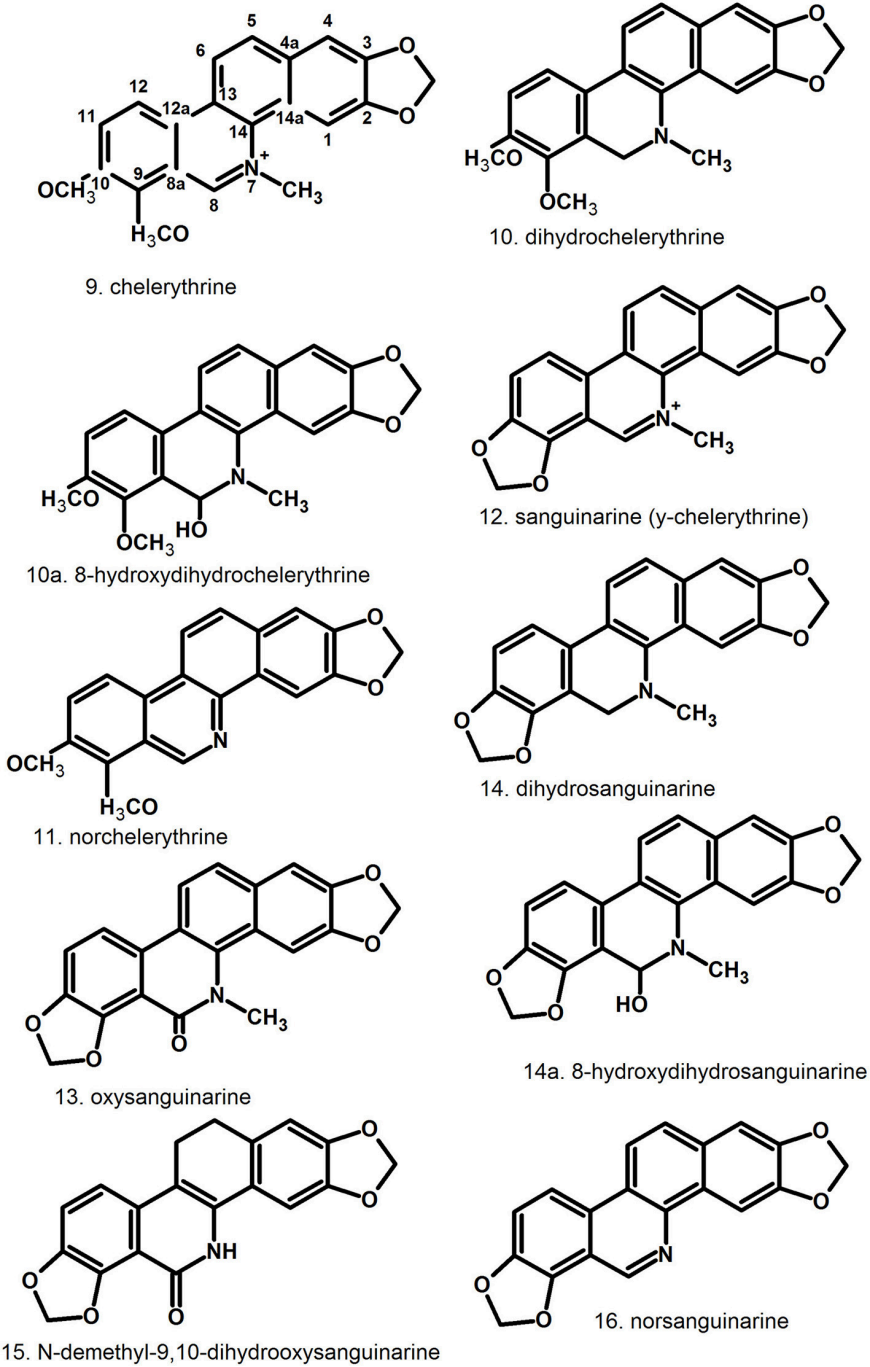
Further structures of phenanthridine (3,4-benzoisoquinoline) alkaloids—chelerythrine [9] and sanguinarine [12] derivatives.

**Figure 3:**
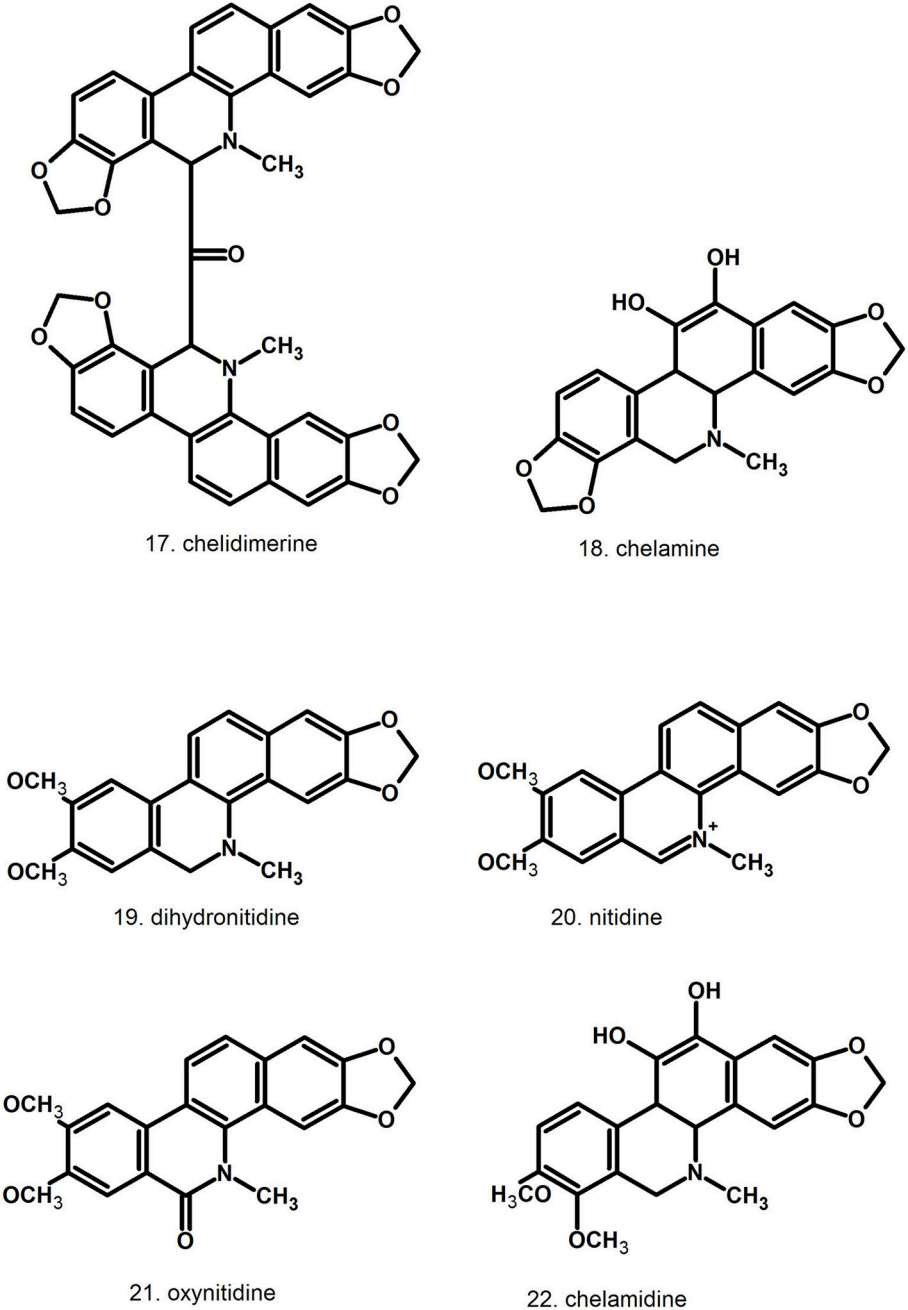
Further structures of minor phenanthridine (3,4-benzoisoquinoline) alkaloids.

**Figure 4:**
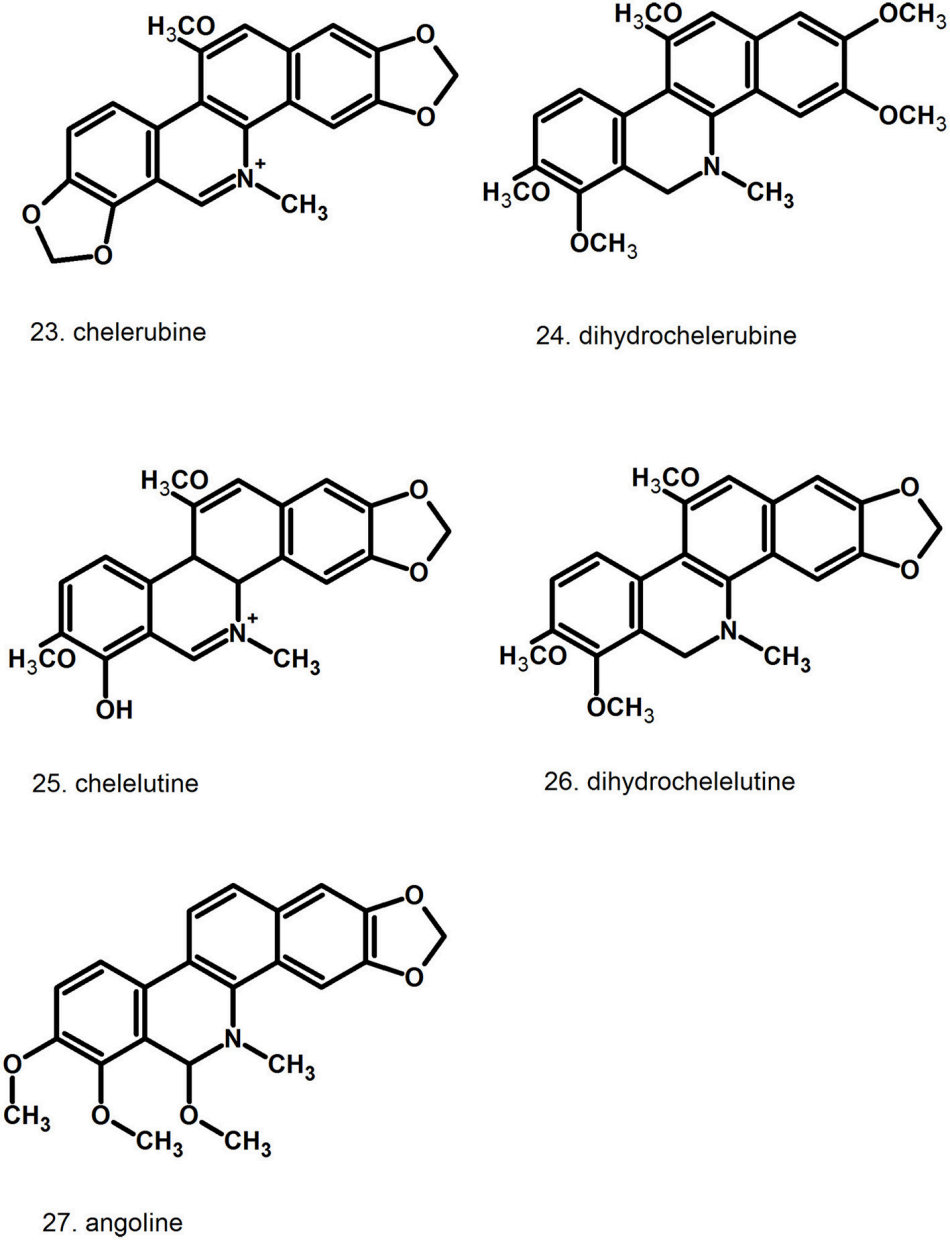
Further structures of minor phenanthridine (3,4-benzoisoquinoline) alkaloids.

**Figure 5:**
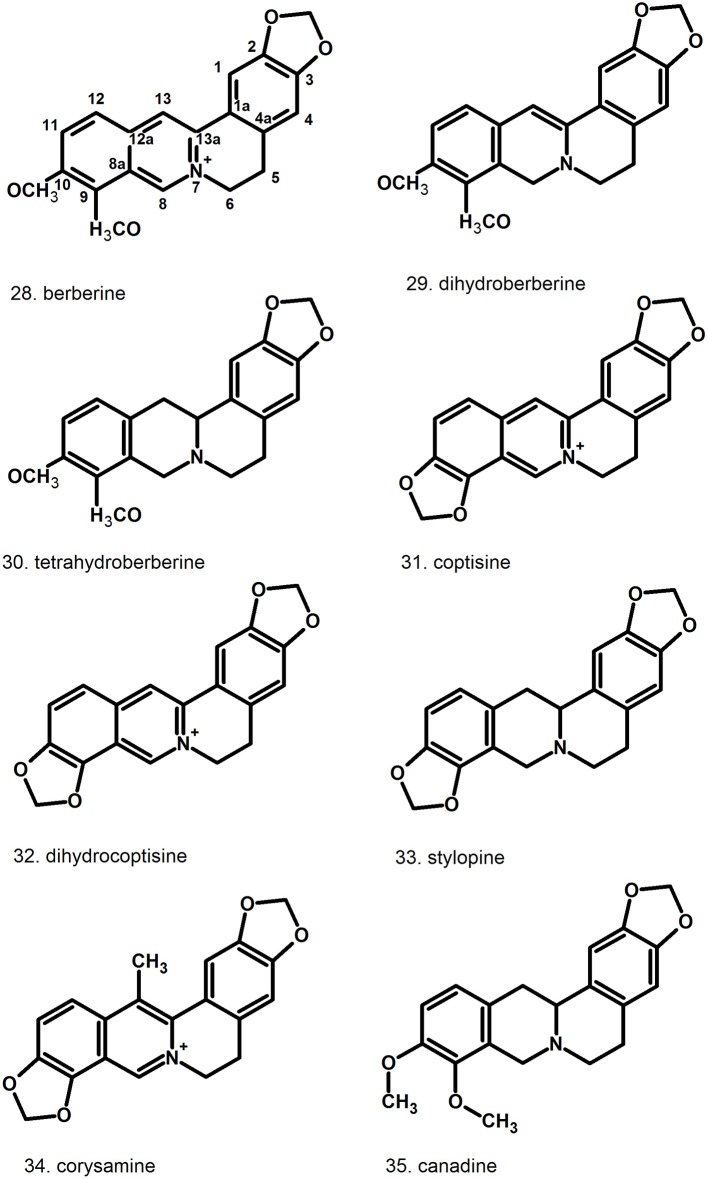
Structures of protoberberine alkaloids.

**Figure 6:**
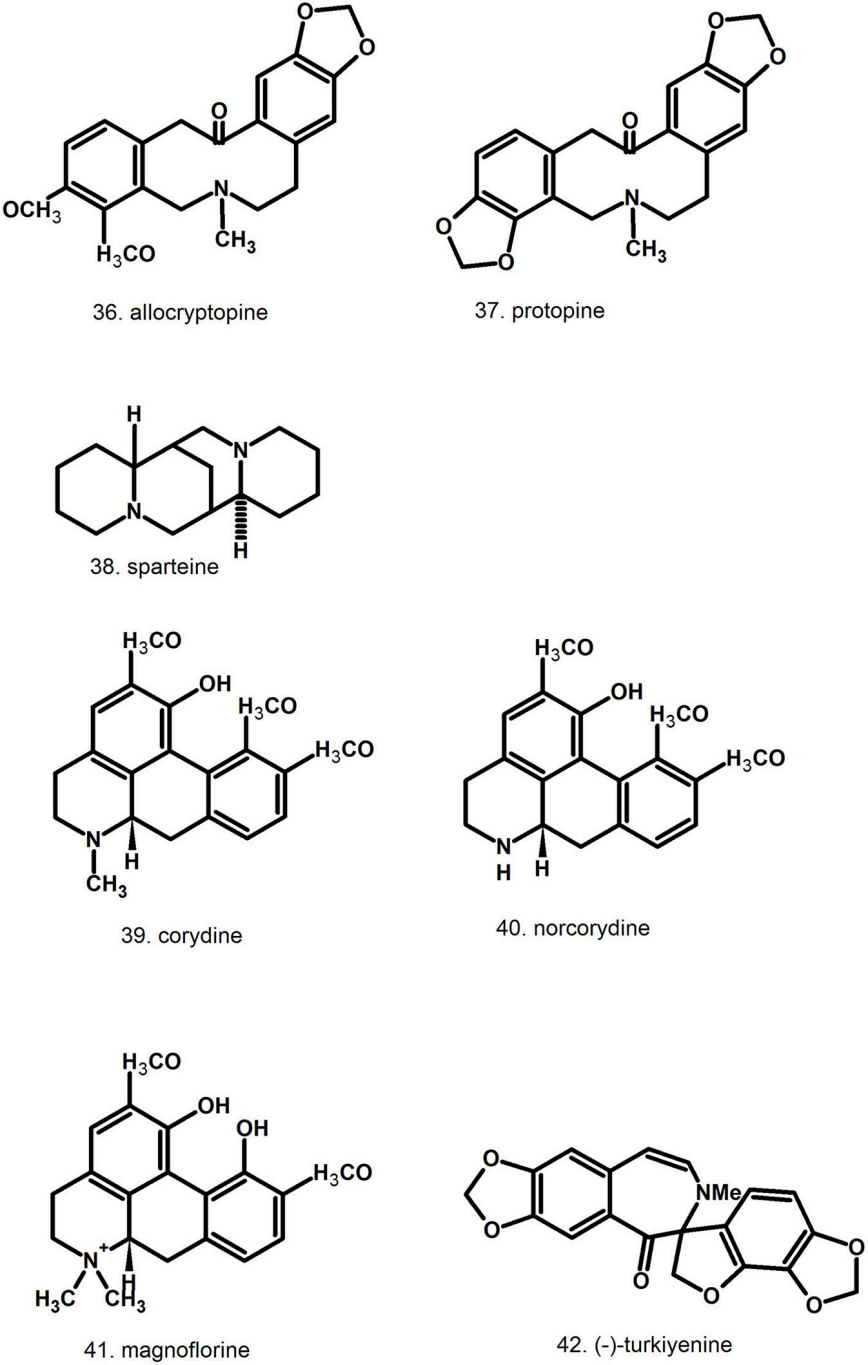
Structures of aporphine, protopine alkaloids, and non-isoquinoline alkaloids.

**Figure 7:**
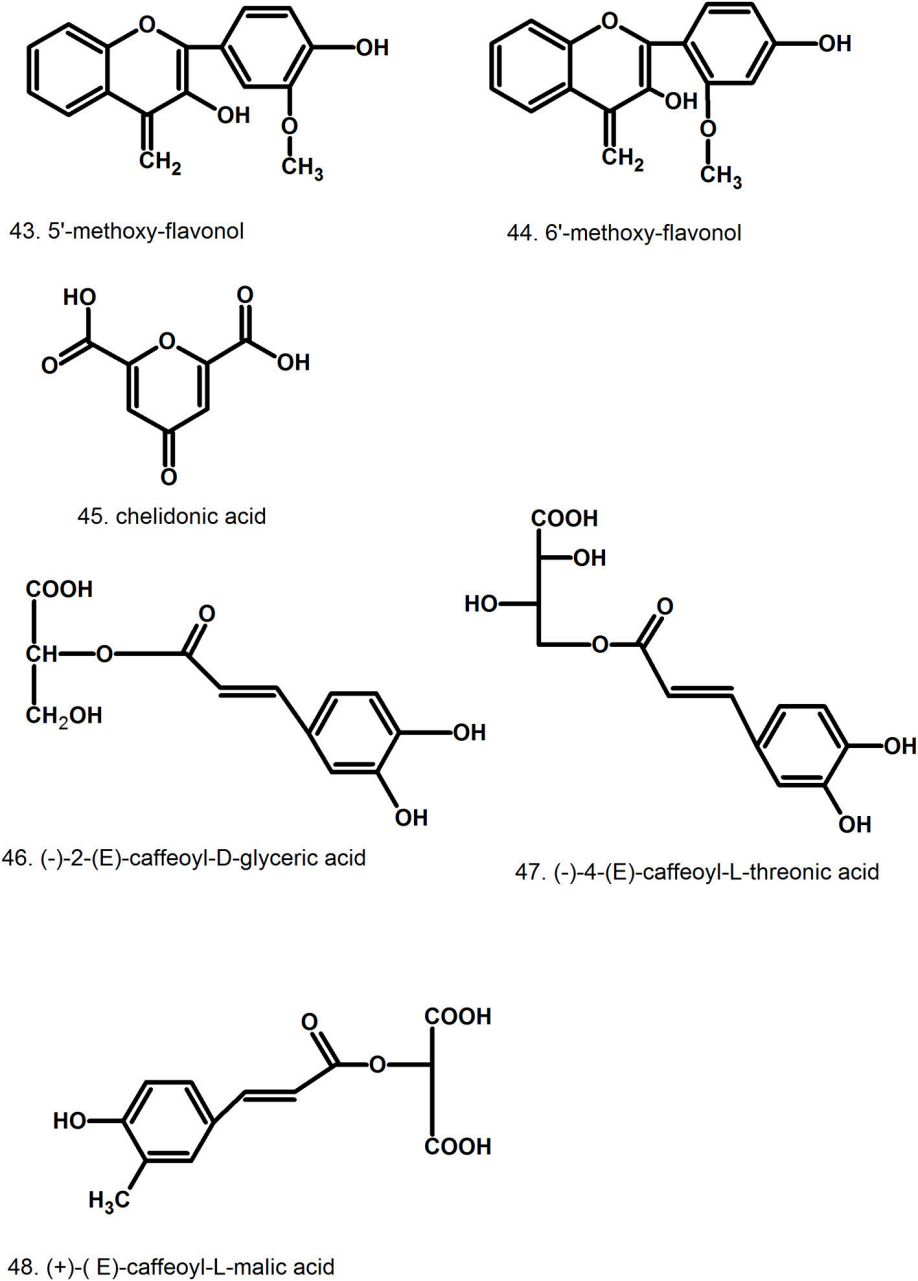
Structures of miscellaneous compounds.

Pharmacologically relevant substances of *C. majus* are isoquinoline alkaloids (Figures 1–7, Table 1). These are the components of latex produced in all plant parts, but flowers. Latex is stored in special secretory cells called laticifers. Presence of articulate laticifers with yellowish content is also used as an authentication microscopic mark in powdered herb by pharmacopoeial monographs. The composition of latex is plant organ specific (Tomē and Colombo, 1995; Sowa et al., 2018). Generally, five groups of alkaloids were found in *C. majus*. These are the derivatives of phenanthridine (3,4-benzoisoquinoline), protoberberine, protopine [37], quinolizidine, aporphine (Kadan et al., 1990, 1992; Pavao and Pinto, 1995; Táborská et al., 1995; Petruczynik et al., 2002; Nečas et al., 2005; Sārközi et al., 2006; Zhou et al., 2012; Kedzia et al., 2013; Grosso et al., 2014; Poormazaheri et al., 2017). More than forty alkaloids of different types were isolated and identified from *C. majus* (Figures 1–7). Major phenanthridine derivatives that were found in aerial and underground parts are (+)-chelidonine [1], chelerythrine [9], (Kadan et al., 1990; Sārközi et al., 2006; Zhou et al., 2012).

**Table 1:** Content [%] of pharmacologically relevant constituents in aerial parts and roots of *Chelidonium majus*.

**Compound**	**Content [% of dry mass]**		**References**
	**Aerial parts**	**Roots**
Chelidonine			Bugatti et al., 1987; Fulde and Wichtl, 1994; Niu and He, 1994; Kedzia et al., 2003; Sārközi et al., 2006; Gañán et al., 2016; Sowa et al., 2018
	t – 0.3	t – 1.51	
Chelerythrine	t – 0.3	t – 0.77	Bugatti et al., 1987; Fulde and Wichtl, 1994; Niu and He, 1994; Shafiee and Jafarabadi, 1998; Sārközi et al., 2006; Gañán et al., 2016; Sowa et al., 2018
Sanguinarine	t – 0.1	0.1 – 0.4	Bugatti et al., 1987; Fulde and Wichtl, 1994; Kedzia et al., 2003; Sārközi et al., 2006; Sowa et al., 2018
Berberine	t – 0.1	t−0.1	Bugatti et al., 1987; Fulde and Wichtl, 1994; Kedzia et al., 2003; Sārközi et al., 2006; Sowa et al., 2018
Coptisine	t – 1.0	t−0.3	Fulde and Wichtl, 1994; Kedzia et al., 2003; Sārközi et al., 2006; Sowa et al., 2018
Flavonols (quercetin, kaempferol and isorhamnetin glycosides)	0.8		Grosso et al., 2014
hydroxycinnamic acids	0.03		Grosso et al., 2014
xanthophylls	0.03; 1.36 (flowers)		Horváth et al., 2010

*t, traces (<0.08%)*.

Protoberberine derivatives that accumulate in higher amounts are coptisine [31], berberine [28], stylopine [33] (Slavik and Slavikowa, 1977; Fulde and Wichtl, 1994; Shafiee and Jafarabadi, 1998; Sārközi et al., 2006). Aporphine alkaloids like corydine [39] also appear (Slavik and Slavikowa, 1977; Shafiee and Jafarabadi, 1998; Kopytko et al., 2005). Two protopines were found in *C. majus*, allocryptopine [36] and protopine [37] (Fulde and Wichtl, 1994; Shafiee and Jafarabadi, 1998; Kopytko et al., 2005). Sparteine [38] is the only representative of quinolizidine alkaloids (Kopytko et al., 2005) but no other publications report its presence. Moreover, new unusual turkiyenine-type alkaloid named (–)-turkiyenine [42] was found in *C. majus* from Turkey (Kadan et al., 1990).

Alkaloid content in different plant organs was found to be unstable (Kustrak et al., 1982; Tomē and Colombo, 1995). Daily variations were probably due to the alkaloid degradation rather than translocation, because of similar time-course of the compounds accumulation in all plant parts. Significant increase of sanguinarine [12], chelerythrine [9], chelidonine [1], and coptisine [31] was observed during the day, with the highest content in the evening, whereupon the alkaloids diminished during the night (Tomē and Colombo, 1995). Day light seems to be the crucial factor influencing alkaloid biosynthesis in *C. majus*, especially in underground parts of the plants (Kustrak et al., 1982; Tomē and Colombo, 1995). Diurnal changes of alkaloid content seem to be less dependent on temperature, what was observed during winter time, when the alkaloid content was low and stable, due to the reduced metabolism and the senescence of the aerial parts. According to Tomē and Colombo (1995) total content of alkaloids in leaves was lower than in underground parts. In latex the content was 32 times higher than in leaves and 9 times higher than in roots. These results suggest that the amount of alkaloids in plant organs depend on the number of laticifers in which they are stored. Moreover, the number of laticifers is probably organ specific. Laticifers in *C. majus* are unbranched (without anastomoses), articulated with perforated transverse cells (Hagel et al., 2008). Articulated laticifers develop from multiple cells. The structures form longitudinal rows composed of series of superimposed cells with perforated end walls. The type of laticifers can differ even within the same plant family. In another *Papaveraceae* species—opium poppy, the perforation of lateral walls leads to the formation of anastomoses (connections) between adjacent laticifer elements, unlike that of Greater celandine (Hagel et al., 2008). From wounded laticifers, a matrix emerges with various organic substances suspended in it. This excretion is called latex and depending on the plant species, it contains proteins, organic acids, alkaloids, sterols, tannins, and mucilage. The growth and development of laticifers runs close to the surrounding phloem, which affects the composition of latex. A major site of alkaloid accumulation in the protoplast of laticifer cells are vesicular organelles, that had been found in opium poppy between early 70's and 80's of the last century (Dickenson and Fairbairn, 1975; Roberts et al., 1983). Unfortunately, research on the site of alkaloid biosynthesis in Greater celandine has not been continued since then. Recent reports concern protein determination in latex and confirm its complex composition (for more information see the separate “Protein subsection” underneath).

The presence and number of laticifers is due to the physiological functions of aerial parts and underground parts of the plant, such as responses to environmental factors, defense against herbivory, metabolic reserves, and energy store (Agrawal and Konno, 2009). Coptisine [31] was found mostly in fruits and herb (Sārközi et al., 2006; Kedzia et al., 2013; Sowa et al., 2018), whereas berberine [28] showed no significant difference between aerial parts and roots (Tomē and Colombo, 1995). Larger amounts of phenanthridine alkaloids like chelidonine [1], chelerythrine [9], sanguinarine [12] were observed in roots rather than in aerial parts of the plants (Tomē and Colombo, 1995; Sowa et al., 2018).

Total alkaloid content (%) in *in vitro* shoots and embryos expressed as chelidonine [1] was 1.53 and 1.58%, respectively (Cirić et al., 2008).

### Phenolic compounds

Several flavonoids were found in aerial parts of *C. majus* in low amounts. Four diglycosides and five monoglycosides were identified as derivatives of kaempferol, quercetin, and isorhamnetin (kaempferol-3-*O*-rutinoside, quercetin-3-*O*-rutinoside, isorhamnetin-3-*O*-glucoside). The identification was based on the mass spectra of the compounds (Grosso et al., 2014). In stems, leaves, and flowers, 5′-methoxy-flavonol [43] and 6′- methoxy-flavonol [44] were also detected (Stancic-Rotaru et al., 2003).

Hydroxycinnamic acids (caffeic, p-coumaric, ferulic) and their derivatives ((–)-2-(*E*)-caffeoyl-_D_-glyceric acid [46], (–)-4-(*E*)-caffeoyl-_L_-threonic acid [47], (–)-2-(*E*)-caffeoyl-_L_-threonic acid lactone, (+)-(*E*)-caffeoyl-_L_-malic acid [48]), as well as hydroxybenzoic acids (genistic, p-hydroxybenzoic) were identified in aerial parts (Hahn and Nahrstedt, 1993; Wojdyło et al., 2007). Later, another three hydroxycinnamic acids were identified using HPLC-DAD-ESI/MS: caffeoyl threonic acid, caffeoyl glyceric acid, caffeoylmalic acid (Grosso et al., 2014), that have been detected previously by Hahn and Nahrstedt (1993). Two caffeoyl acid derivatives isomers with precursor ions at *m/s* 359 corresponding to rosmarinic acid were also found in aerial parts (Grosso et al., 2014).

### Proteins

A phytocystatin—chelidocystatin was one of the first proteins isolated from latex and characterized (Rogelja et al., 1998). Cystatins, a class of thiol protease inhibitors are involved in defense and stress-response mechanisms and could also contribute to the antimicrobial and antiviral activity of *C. majus* latex (Benchabane et al., 2010). Whether or not the presence of cystatin is relevant to the medicinal properties and such traditional folk uses as anti-warts is yet to be found out.

In a series of papers, Nawrot et al. (2007a,b, 2008, 2013, 2014, 2016, 2017a,b) described a number of proteins from root and leaf latex. Proteomic analysis using LC-ESI-MS/MS system revealed the presence of three categories of proteins according to their functions: proteins involved in disease and defenses responses (i.e., superoxide dismutases, lactoylglutathione lyases), nucleic acid binding proteins (i.e., glycine-rich proteins, nucleic acid binding, DNA-binding, or RNA-binding proteins), and these that are involved in general metabolism (acyl-CoA binding protein, malate dehydrogenase, flavodoxin-like quinone reductase, ubiquitin, polyubiquitin, serine/threonine protein kinases, rubber elongation factor). A total of 21 proteins were identified in *C. majus* latex, although in several cases the identification was based on correlation between experimental and the theoretical p*I*/molecular mass, due to their low score results. The results shown less complexity of latex proteins in this species compared to opium poppy. Their contribution to the traditional use of *C. majus* as antiviral and antimicrobial remedy has to be further explored and may, in combination with highly active alkaloids, render unique synergistic effects providing a multifaceted tool for combat against troublesome infections. In living plants, these proteins are probably also serving as a chemical defense against pathogens (Nawrot et al., 2007a,b, 2014, 2017a,b). Protein-bound polysaccharide (CM-AIa) bearing immunomodulatory and cytotoxic activity was isolated by Song et al. (2002).

### Other compounds

Organic acids: chelidonic [45], malic, citric, succinic, (Kopytko et al., 2005); Biogenic amines: histamine, methyloamine, tyramine (Kwasniewski, 1958); choline in fruits (Kwasniewski, 1958); essential oil constituents (Hansel et al., 1992; Kohlmünzer, 2000) triterpenoids (Hahn and Nahrstedt, 1993; Deng et al., 2016); saponins (Kwasniewski, 1958; Kopytko et al., 2005); Resin (Hahn and Nahrstedt, 1993); vitamins A, C, nicotinic acid (Hahn and Nahrstedt, 1993; Kopytko et al., 2005).

Flowers contain xanthophyll pigments like lutein, violaxanthin, flavoxanthin, chrysanthemoxanthin (Horváth et al., 2010).

## Methods for analysis of active components from *Chelidonium majus*

### Alkaloids

First isolation of *Chelidonium* alkaloids was achieved in nineteenth century with an important contribution from Mr. Emanuel Merck's company at Darmstadt (Henschke, 1888; Schmidt, 1888) and obtaining and characterization of pure compounds (chelidonine [1], chelerythrine [9], protopine [37]) was successful in the following years (Selle, 1890; Wintgen and Schmidt, 1901). Alkaloids of *C. majus* occur as salts or bases depending on pH of medium; thus, their extraction was mostly carried out in acidic condition to convert all compounds to water-soluble salts. Methanol or ethanol often with addition of water (Bugatti et al., 1987; Han et al., 1991; Koriem et al., 2013) and hydrochloric (Kursinszki et al., 2006; Gu et al., 2010) or acetic acid (Paulsen et al., 2015) were used as extractants. The isolation from plant material was also achieved with the use of pure acidified water; further, the solution was alkalized with ammonia or sodium hydroxide to obtain base forms followed by liquid-liquid extraction with organic solvents such as dichloromethane, butanol or chloroform (Sārközi et al., 2006; Sárközi et al., 2007; Migas et al., 2012; Jesionek et al., 2016; Bogucka-Kocka and Zalewski, 2017). The isolation was conducted by percolation (Capistrano et al., 2015), maceration (Koriem et al., 2013; Borghini et al., 2015), heating under reflux (Gu et al., 2010; Yao et al., 2011; Seidler-Łozykowska et al., 2016), in water bath (Sārközi et al., 2006) or Soxhlet apparatus (Bugatti et al., 1987; Stuppner and Ganzera, 1995) as well as ultrasound assisted (UAE) (Kursinszki et al., 2006; Sárközi et al., 2007; Paulsen et al., 2015; Jesionek et al., 2016) or microwave energy (MAE) (Then et al., 2000; Zhou et al., 2012). Supercritical fluid extraction (SFE) (Then et al., 2000) or SFE combined with enhanced solvent extraction (ESE) and low pressure solvent extraction with water (LPSE) (Gañán et al., 2016) was also applied. Moreover, *in situ* solvent formation microextraction (ISFME) with the use of ion-pairing agent (KPF6) and a water-miscible ionic liquid ([C6MIM][Br]) which formed a hydrophobic ionic liquid extraction phase ([C6MIM]PF6) for the pre-concentration of sanguinarine [12] and chelerythrine [9] was elaborated by Wu and Du (2012). After extraction, the solution was usually filtrated through a 0.22-μm membrane (Gu et al., 2010) or additionally purified using solid phase extraction (SPE) on C18 cartridge (Stuppner and Ganzera, 1995; Kursinszki et al., 2006; Sārközi et al., 2006). Sārközi et al. (2006) developed ion-pair SPE with n-heptanesulfonic acid (HS). Dried residue obtained after extraction was dissolved in methanol with 0.05 M hydrochloric acid, diluted with 0.05 M aqueous solution of HS and loaded on an SPE C18 microcolumn. Further, 70% HS (0.05 M) in methanol was used to remove the matrix and 5% HS (0.05 M) in methanol to elute analytes. The examples of conditions used to isolate alkaloids from *C. majus* are presented in Supplementary Table 1.

### Thin layer chromatography (TLC)

TLC was mostly employed for screening purposes or for qualitative analysis of alkaloid composition in *C. majus* extracts. This technique has been largely limited to the screening and multiple sample fingerprinting, but usually does not enable high-sensitivity or high-resolution insight into the minor components of the phytochemical profile. Despite of being less intensively modernized in comparison to column-based techniques like (U)HPLC, this method is still favored when cost-effectiveness and simplicity of sample preparation is important, for example in herbal industries and educational use. Some recent developments in mass spectrometry hyphenation in form of matrix transfer or DART (direct analysis in real time) used already in studying of other species (Móricz et al., 2018) should be useful also for *C. majus* alkaloids.

Silica was the most common stationary phase, often impregnated with salts (Ni, Zn, Cr, Co) to improve the selectivity (Wagner et al., 1984; Then et al., 2000; Waksmundzka-Hajnos et al., 2000; Sārközi et al., 2006; Sárközi et al., 2007; Petruczynik et al., 2008; Jesionek et al., 2016) Silica modified with octadecyl (C-18) and cyanopropyl (CN) groups and aqueous solutions of methanol or isopropanol with ammonia or diethylamine which prevented tailing of chromatographic bands were sporadically applied (Petruczynik et al., 2007). Petruczynik et al. (2008) combined various types of stationary phases e.g., cyanopropyl silica with silica or octadecyl silica to obtain adsorbent gradient.

Apart from the routine isocratic elution with solvent mixtures, using different modes of gradient elution improved the separation, e.g., as a two-dimensional or unidimensional multiple development (Szumiło and Flieger, 1999; Waksmundzka-Hajnos et al., 2000; Migas et al., 2012), bivariant multiple development (Bogucka-Kocka and Zalewski, 2017), or stepwise gradient elution (Matysik and Jusiak, 1990; Waksmundzka-Hajnos et al., 2000; Bogucka-Kocka and Zalewski, 2017). TLC separation of alkaloids was supported by use of magnetic field (Malinowska et al., 2017) or forced flow of mobile phase (OPLC—overpressure layer chromatography or optimum performance laminar chromatography; Pothier et al., 1993; Malinowska et al., 2005).

TLC was also applied for direct bioautography (TLC-DB) to test the antibacterial activity of *C. majus* extracts (Sárközi et al., 2007; Móricz et al., 2015; Jesionek et al., 2016) combined with densitometry for quantitative analysis (Then et al., 2000; Sārközi et al., 2006) and it proved useful as preparative technique for isolation of alkaloids (Waksmundzka-Hajnos et al., 2002; Koriem et al., 2013).

### High performance liquid chromatography (HPLC)

High performance liquid chromatography (HPLC) and ultra-fast liquid chromatography (UFLC) have been the most often applied analytical techniques. For many years, separation of *C. majus* extracts was mostly carried out in reversed phase (RP) system using long (150 or 250 mm) C18 columns with 4.6 mm of diameter and 5 μm of particle size (Niu and He, 1994; Petruczynik et al., 2002; Kursinszki et al., 2006; Borghini et al., 2015; Paulsen et al., 2015; Gañán et al., 2016). More recently, adsorbents with smaller particles (≤ 3 μm) or shorter columns (Prosen and Pendry, 2016; Seidler-Łozykowska et al., 2016) were also applied to achieve shorter separation and saving solvents. Mobile phases are usually composed of water and acetonitrile or/and methanol with various additives e.g., ammonium formate/acetate (Borghini et al., 2015; Seidler-Łozykowska et al., 2016), organic amines (triethyl-, tetrabutylamine) (Paulsen et al., 2015), ion-pair reagents (sodiumdodecylsulfate, alkylsulfonic acids) (Gañán et al., 2016). The additives were necessary to reduce peak tailing forming as a results of interaction of alkaloid cationic forms with residual silanol group of stationary phase. Eluents were acidified with acetic or formic acid to pH < 4 to avoid the co-occurrence of ionic and uncharged forms.

Normal-phase (NP) chromatography set ups were used rather seldom in analysis of alkaloids. Here, silica columns was eluted with sodium acetate in methanol, dioxane and acetic acid mixture (Bugatti et al., 1987) or chloroform and methanol with trifluoroacetic acid (Rey et al., 1993) Also, cyanopropyl stationary phase was eluted with acetonitrile, tetrahydrofuran, dioxane or methanol with phosphate buffer and octane-1-sulfonic acid sodium salt or di-(2-ethyl hexyl) orthophosphoric acid (HDEHP) (Petruczynik et al., 2002).

Alkaloids have a strong absorption in UV region and have ability to fluorescence; thus both spectrophotometric and fluorescence detector (Wu and Du, 2012) were applied. Additionally, ESI-MS and/or NMR were coupled with HPLC to confirm the identity and structure elucidation of new alkaloids (Paulsen et al., 2015). Moreover, preparative separation of particular alkaloids from *C. majus* extract using silica gel and sequentially elution with petroleum ether, ethyl acetate and methanol (Yao et al., 2011) or CaCO_3_ and different compositions of toluene-hexane and acetone-hexane mixtures (Horváth et al., 2010) was performed with use of LC system.

### Capillary electrophoresis CE

CE method with spectrophotometric (DAD) and fluorescence detection (UV-LEDIF ultraviolet light-emitting diode-induced native fluorescence or LED-fluorescence) was also used for *C. majus* analysis (Stuppner and Ganzera, 1995; Ševčik et al., 2000; Kulp et al., 2011; Zhou et al., 2012; Kulp and Bragina, 2013); however, this technique has not yet won high popularity and it is also limited to charged or polar compounds. Recently, Sun et al. (2015) elaborated the microchip variant of CE with laser-induced fluorescence detection and 50% formamide as a run buffer and this technique was applied for separation of chelerythrine [9] and sanguinarine [12]. The examples of application the separation techniques in analysis of alkaloids from *C. majus* are presented in Supplementary Table 2.

### Spectrophotometry

Spectrophotometric method given in European Pharmacopeia (monograph of Greater celandine) may be useful to estimate of total alkaloids in *C. majus* extract. The sample is mixed with sulphuric and chromotropic acid, and heated 10 min. at 100°C in water-bath. After cooling to 20°C, the absorption of sample is measured at 570 nm and the amount of alkaloid is expressed as chelidonine [1]. This approach was applied by (Then et al., 2000; Seidler-Łozykowska et al., 2016). However, for research purposes, a total-content approach should be discouraged as too inaccurate and sometimes misleading about contribution of each component of an actual alkaloid profile.

In summary, most of the published preparative and analytical approaches were rather routine and typical for phytochemical studies. However, the efficiency of standard methods often appeared to be insufficient. The properties that influenced the separation and analysis processes depend mostly on the tertiary or quaternary character, oxygenation and secondary cyclization pattern. Among the most important features are thermo- and photo-sensitivity, so the procedures should be carried out in mild conditions which has not always been considered. Thus, due to the diversity in physicochemical properties, even among compounds from a single subclass, various specialized modifications were applied which in most cases significantly improved the separation.

Another issue that should be addressed in future analytical studies is that numerous minor alkaloids have been frequently missed or overlooked whereas some of them have profound activity. The focus should be on comprehensive qualitative and quantitative profiling under non-destructive conditions that would reveal the full phytochemical complexity and allow to understand the intraspecific and environmental variation of *C. majus*.

### Other components

#### Carotenoids

Carotenoids were determined in aerial parts (Varzaru et al., 2015) and flowers (Horváth et al., 2010) of greater celandine. The components were hydrolyzed with alcoholic solution of potassium hydroxide and extracts were analyzed on RP-HPLC using C18 column in isocratic mode using 13% of water in acetone (Varzaru et al., 2015) or in gradient mode with mixture of three eluents: 12% (v/v) water in methanol (A), methanol (B), and 50% (v/v) acetone in methanol (C) in different proportions (Horváth et al., 2010).

#### Phenolic compounds

Grosso et al. (2014) tested different combinations: time, temperature type of extraction, and eluent composition and used Box–Behnken design to establish the effective extraction conditions for phenolic compounds. 76.8% of methanol, 150.0 min and 60°C were found to be optimal. Further, hydroxycinnamic acids and flavonoids were characterized by HPLC-DAD-ESI/MS^n^ (C18 column: 150 × 1.0 mm, 3 μm) and quantified using RP-HPLC DAD (C18 column: 250 × 4.6 mm, 5 μm). For both methods, gradient elution with mobile phase composed of 1% acetic acid in water and methanol was applied (Grosso et al., 2014). Hence, the method for phenolic analysis is similar to some which were used for alkaloids and there would be reasonable to include phenolic compounds in any future HPLC or LC-MS profiling of this herb.

#### Proteins

The composition of proteins in *C. majus* milky sap was studied by Nawrot et al. (2007a,b, 2014, 2017a,b). The compounds were pre-separated using two-dimensional gel electrophoresis and further, HPLC on BEH C18 column (100 mm × 100 μm, 1.7 μm particle diameter) (Nawrot et al., 2017b) or nano-HPLC on 75 μm analytical column (Nawrot et al., 2007a, 2014, 2017a) and gradient elution with mobile phase containing 0.1% (v/v) formic acid in water and in acetonitrile were performed. Proteins were identified by tandem mass spectrometry (MS/MS).

#### Micro and macro-elements

Mineral composition of greater celandine herb and root, aqueous solutions (infusion, decoction) and alcoholic extracts were determined with the use of inductively coupled plasma atomic emission spectrometry (Then et al., 2000; Sárközi et al., 2005).

## Pharmacological activities and clinical evidence

Obviously enough, the great majority of published pharmacological properties of *C. majus* pertains to the complex mixture or individual alkaloids. Recently, more and more attention has been also paid to proteins from latex, that could significantly contribute to the observed activities. Other compounds, such as various phenolics and chelidonic acid were rarely considered. The versatile pharmacological activities of coptisine [31], one of the major constituents of *C. majus* as well as the less abundant berberine [28], have been widely described in the literature but usually as compounds obtained from other sources, such as *Coptis* (*Ranunculaceae*) (Tan et al., 2016) or *Berberidaceae* (Imanshahidi and Hosseinzadeh, 2008).

However, these compounds are also contributing significantly to many of the pharmacological properties of *C. majus*.

From the growing evidence obtained both *in vitro* and *in vivo*, with several examples of *ex vivo* studies on isolated organs, it is clear that four types of medicinal properties are predominating: antimicrobial and antiviral, hepatic and gastric, anti-inflammatory, and finally anticancer. Several other activites have been also reported but less extensively.

### Activites *in vitro* and *in vivo*

#### Antibacterial and antifungal

The antimicrobial activity of *C. majus* is attributed mostly to the alkaloids and flavonoids (Zuo et al., 2008). This kind of activity was reported already in the early research on chelidonium alkaloids, e.g., by Stickl (1928) who proved the bactericidal properties against Gram-positive strains (*Staphylococcus aureus* and *Bacillus anthracis*) with chelerythrine [9] and sanguinarine [12] being more potent than chelidonine [1] and berberine [28].

In experiments with multidrug resistant bacteria existing in surgical wounds and infections of critically ill patients, *C. majus* ethanol extract affected Gram-positive bacteria. Ethanolic extracts of *C. majus* also showed antimicrobial activity against *Bacillus cereus, E. coli, Pseudomonas aeruginosa, S. aureus*, (Kokoska et al., 2002). The complex composition of alkaloids can manifest wide spectrum of antimicrobial activity, arising from different chemical structures of the compounds. Hence, antimicrobial activity of *C. majus* was also tested with a use of different solvent extraction. Antibacterial and antifungal tests were performed using 96% methanol extracts from leaves and petioles of plants grown in nature as well as *in vitro* shoots and embryos (Cirić et al., 2008). Methanolic extracts were examined against Gram-positive (*Bacillus subtilis, Micrococcus luteus, Sarcinia lutea*, and *S. aureus*), Gram-negative bacteria (*E. coli, Proteus mirabilis, Salmonella enteritidis*), plant pathogens—*Agrobacterium rhizogenes, A. tumefaciens*), and clinically isolated *C. albicans*. Both, *in vivo* and *in vitro* derived plant material extracts showed similar bioactivity, with a slight advantage of *in vitro* shoots. Only few extracts were equally active (80 mg/ml) against *E. coli, S. enteritidis*, and *C. albicans*, when compared to the commercially available antibacterial and antifungal drugs (streptomycin, bifonazole, respectively), while the rest of them showed low or no activity (Cirić et al., 2008).

Apart from plant extracts, as it was previously summarized by Kedzia and Hołderna-Kedzia (2013), individually tested compounds showed different antimicrobial activity. Chelerythrine [9] and sanguinarine [12] were significantly more potent than chelidonine [1] against Gram-positive (*S. aureus, S. epidermidis, B. subtilis, B. anthracis*), Gram-negative (*P. aeruginosa, E. coli, Klebsiella pneumoniae, Salmonella gallinarum, S. typhi, S. paratyphi, Proteus vulgaris, Shigella flexneri*), and acid-fast mycobacteria (*Mycobacterium tuberculosis, M. smegmatis*). Moreover, chelerythrine [9] exhibited antimicrobial activity against *S. aureus* and *M. smegmatis*, while chelerythrine [9] derivatives: 8-hydroxydihydrosanguinarine [14a], 8-hydroxydihydrochelerythrine [10a], dihydrosanguinarine [10], and dihydrochelerythrine [14] were active against methicillin-resistant *S. aureus* (MIC_50_ = 0.49, 0.98, 23.4, 46.9 μg/ml, respectively). The strongest activity was presented by 8-hydroxydihydrosanguinarine at MIC_90_ = 1.95 μg/ml, comparable to that of vancomycin (2.23 μg/ml) (Zuo et al., 2008). In the ethanol extract of *C. majus* aerial parts there are several other alkaloids that show potent antimicrobial inhibitory effect. For instance, berberine [28] was effective against Gram-negative bacteria—*Vibrio cholerae* and *E. coli*. It damaged bacterial fimbria, thereby inhibited adhesion to the mucosal surface (Imanshahidi and Hosseinzadeh, 2008; Wongbutdee, 2009).

Enzymes included in latex, like extracellular peroxidases, DNases, and lectin-like-active glycoproteins can also exhibit antimicrobial activity. Moreover, chelidocystatines protect plant against pests and are among the components of latex which presumably contribute to removal of warts resulting from human papilloma virus infection (Rogelja et al., 1998; Song et al., 2002; Gerenčer et al., 2006; Nawrot et al., 2007b; Cirić et al., 2008).

The antifungal activity was tested with the use of plant latex, and different solvent extracts from aerial and underground parts like ethanol/methanol ether, chloroform, acetone, and water (Kokoska et al., 2002; Kedzia et al., 2003). Chelerythrine [9], sanguinarine [12], chelidonine [1], and their derivatives were also individually tested against large number of human and plant pathogenic fungi, i.e., *Aspergillus fumigatus, A. niger, Candida* sp. *Cladosporium herbarum, Cryptococcus neoformans, Epidermophyton floccosum, Fusarium* sp. *Keratinomyces ajelloi, Microsporum* sp. *Penicillium notatum, Rhodotorula rubra, Scopulariopsis brevicaulis, Torlopsis utilis, Trichophyton* sp. The effect of *C. majus* extracts on pathogenic fungi was significantly weaker compared to the effect on pathogenic bacteria. For example, the MIC of ethanol and methanol extracts was ranging between 1.5 and 8 mg/ml against the most resistant bacteria, whereas the MIC of ethanol herb extract against the most pathogenic fungi was 20–40 mg/ml (Pepeljnjak et al., 2003). Chelerythrine [9], sanguinarine [12] and their derivatives (e.g., 8-hydroxydihydrochelerythrine [10a]) were up to several times stronger than chelidonine [1] (Ma et al., 2000; Meng et al., 2009; Kedzia and Hołderna-Kedzia, 2013). Chelerythrine [9] was also able to inhibit spore germination of several plant pathogenic fungi: *Sphaerulina juglandis, Septoria microspora, Fusarium oxysporum*, and *Curvularia lunata*. It suggests the actual biological—antipathogenic function of isoquinoline alkaloids in the plant (Wei et al., 2017). However, no particular structural feature seems to be pinpointed as a determinant of antimicrobial potency despite some variation in activity toward different strains between individual alkaloids. It is rather the combination of them and other compounds targeting multiple sites of action that result in the observed final effect. This hypothesis still needs experimental verification for potential synergies or other interactions.

#### Antiviral

The glycosaminoglycan present in the latex inhibits intracellular human immunodeficiency virus HIV viral migration and blocks reverse transcriptase (Gerenčer et al., 2006). Moreover, individually tested five *C. majus* alkaloids: chelidonine [1], chelerythrine [9], sanguinarine [12], coptisine [31], and berberine [28] were able to inhibit the development of HIV-1. The first two decreased the activity of the virus reverse transcriptase at the concentrations 150–200 μg/ml, while sanguinarine [12], berberine [28], and coptisine [31] were already active at concentrations of 50–150 μg/ml (Tan et al., 1991).

The chloroform extract in the concentration of 35 μg/ml decreased the number of adenoviruses responsible for inducing acute fevelitis of the upper respiratory tract and conjunctiva in humans (Kéry et al., 1987). The experiments with animals showed that ethanol extract of *C. majus* inhibited encephalomyocarditis virus in 45% of experimental mice, whereas berberine [28] tested in the concentration range between 20 and 125 μg/ml inhibited influenza virus type A and B in chicken embryos with 33–99.97% efficiency (data previously reviewed by Kedzia et al., 2003). These results were presented only once, and from that time have never been confirmed or repeated.

#### Antiprotozoal

Herb and root water extracts, as well as sanguinarine [12] (2–4 μg/ml) were highly effective in the treatment of trichomoniasis caused by *Trichomonas vaginalis*. After 8–10 days of treatment there was no protozoa detected in genitals of nearly 64% young girl patients. Sanguinarine [12] was also found to inhibit the development of *Entamoeba histolytica*, responsible for the hepatic abscess (Kozicka and Radomanski, 1963; Vychkanova et al., 1969).

#### Liver and biliary tract

One of the most widespread and repeatedly mentioned indications of *C. majus*, both in European/Mediterranean and East Asian (TCM) tradition, was for various liver complaints. It may date back to “*signatura rerum”* rule from coloration of the latex and flowers but obviously must have been verified by observations. Nowadays, even though this indication has been supported by just a few *in vitro* and *in vivo* studies, caution is necessary for alleged hepatotoxicity. Also, clinical evidence is not sufficient to ultimately recommend this herb and galenic preparations thereof (European Medicines Agency, 2011).

Some of the hepatoprotective and choleretic/cholagogue activity could be more aptly attributed to the presence of hydroxycinnamic (caffeic) acids esters which have been quite frequently overlooked in alkaloid-focused studies (Weiskirchen, 2016).

The question whether the supposed stimulation of bile flow is caused only by cholagogue activity or also by increasing bile production or excretion was first addressed by Rentz (1947). Comparison of guinea pigs and rats (that do not have gall bladder and did not respond to the treatment) reaction to *C. majus* tincture suggested only cholecystokinetic mechanism of action, attributed by the author to the stimulation of smooth musculature by berberine [28]. However, the tincture composition was unknown.

Vahlensieck et al. (1995) indicated using isolated rat livers that beside the earlier reports on cholecystokinetic action, also increase of bile production contributes to the final outcome. The activity was not very high, reaching 20% increase by perfusion with *C. majus* extract. The activity of alkaloid and polyphenol fractions separately were only about half of that. It suggests an additive action of the complex mixture of all active constituents.

The antispasmodic activity of *C. majus* extract was tested in trials based on acetylcholine (ACh)-induced contraction in isolated rat ileal smooth muscle (Boegge et al., 1996). The extract was found to be moderate antagonist (12.7%; 2.0 × 10^−4^ g/ml organ bath) against (ACh)-induced contraction compared to caffeoylmalic acid [48] (6.9%; 2.5 × 10^−5^ g/ml) and coptisine [31] (16.5%; 1.0 × 10^−5^ g/ml). Also, individual alkaloids, i.e., chelidonine [1], stylopine [33], and coptisine [31] have been tested for relaxant activity on ileum smooth muscles (Hiller et al., 1998). Among them, chelidonine [1] and stylopine [33] showed papaverine-like musculotropic action, whereas coptisine [31] was ineffective in BaCl2 stimulation model. In carbachol and electric field induced contractions, all three alkaloids and ethanolic extracts were effective.

In a different contraction model—isolated and perfused porcine uterus, the commercial alcoholic extract exhibited two-phase response, initially stimulating very strong contractions followed by a longer relaxation period (Kuenzel et al., 2013). These properties were suggested as potentially useful in supporting artificial insemination or facilitating fertilization by acceleration of sperm movement toward fallopian tubes. However, this indication was rather unknown in traditional usage and would be a novel application of *C. majus*.

#### Cytoprotective

The macerated ethanol extract from juice expressed from pulped fresh plant material (according to the homeopathic recipes) was able to counteract carbon tetrachloride induced hepatotoxicity (Mitra et al., 1996). The effects of extract administration included reduced cell necrosis, absence of fibrosis, and lower lipid accumulation. Here again, no reliable data on the composition of the tested extract was available. *C. majus* primary tincture (German Homeopathic Pharmacopeia), diluted 100x and 1,000x was significantly effective against cadmium-induced hepatoxicity in HepG2 and primary rat hepatocyte models (Gebhardt, 2009). The activity was stronger than the proprietary compound preparation and similar to the recognized hepatoprotective herb—*Silybum marianum*. The putative mechanisms of cytoprotective activity was associated to oxidative stress relieve as demonstrated by improvement of several parameters such as lipid peroxidation, intracellular Reactive Oxygen Species, reduced glutathione (GSH) level, as well as diminished apoptosis symptoms (nuclei fragmentation, cytochrome C release, caspase 3 activation). Hepatotoxicity caused by cadmium was also ameliorated *in vivo* in mice and *ex vivo* in hepatocyte cultures. In mice, administration of 50 or 75 mg/kg body weight chelidonine [1], also in form of nanocapsules, improved histopathological picture of livers damaged by Cd treatment. Also, biochemical parameters such as ALT, AST, and ALP activities were lowered to the levels intermediate between control animals and Cd treated ones. Moreover, the expression of cell death related genes Bax and Bcl-2 was modulated to the levels closer to the Cd untreated animals. In all tested hepatotoxicity parameters, nanoencapsulated chelidonine [1] was more efficient. It was corroborated with the ca. 1/3 higher distribution of chelidonine [1] from nanoformulation into the liver tissues. The putative mechanisms relate to alleviation of oxidative stress as revealed by improvement of antioxidant status (lower lipid peroxidation, higher GSH level, and SOD and CAT activities) and various cell death and inflammation markers (decreased protein level of TNF-α, IL-6, NFκB, p65, cas-3, iNOS) (Paul et al., 2013).

Notwithstanding, the literature evidence supporting beneficial properties in hepatobiliary disorders from both *in vitro, in vivo*, or *ex vivo* studies is still less abundant than case reports on liver toxicity. This, quite surprising disparity, should motivate pharmacologists and clinical researchers to do further and more insightful studies to explain the mechanisms of action and pinpoint the most active constituents or their combinations.

#### Antiproliferative, pro-apoptotic, and cytotoxicity to cancer cell lines

As in case of liver and biliary tract disorders, the antitumor properties have been indicated since antiquity. Today, this kind of activity belongs to the most intensely investigated. Unlike the hepatoprotective properties, this kind of properties has been quite well-documented in a high number of studies. Mostly, some of the major alkaloids are expected to be able to cause cell death or stop proliferation of cancerous cells. This is based on the ability of berberine [28], chelerythrine [9], sanguinarine [12], and to some extent also other alkaloids to intercalate DNA that should interfere with replication and cell division (Philchenkov et al., 2008; Basu et al., 2013; Noureini et al., 2017). However, other mechanisms have been also discovered, albeit most studies used *in vitro* experiments on cell lines. Chelidonine [1] appeared to exert its cytostatic activity through interactions with microtubules and thereby causing cell cycle arrest (Panzer et al., 2001; Havelek et al., 2016a).

The selected examples of cell line-based studies on cytotoxic properties of *C. majus* and its major alkaloids are summarized in Table 2.

**Table 2:** Influence of *C. majus* and its alkaloids on various cell lines *in vitro*.

**Cell line**	**Tested extracts/compounds**	**Main observed outcome/response**	**References**
HaCaT—human keratinocyte	Dry extract-−0.68% alkaloids, pure berberine [28], chelidonine [1], chelerythrine [9], hydrastinine, sanguinarine [12]	Antiproliferative activity with IC_50_ lowest for sanguinarine (2.26 μM), extract (as chelidonine) ca. 5.68 μM, chelidonine and chelerythrine ca. 28 μM, low activity of berberine and hydrastinine;	Vavrečková et al., 1996
WHCO5 -squamous esophageal cancer, HeLa, Vero, 293Graham, HS-27 -transformed human foreskin fibroblasts	Chelidonine [1] 10-134 μM	Cytotoxic to all types of cell lines, little specific to malignant cells (HeLa, WHCO5), mitotic arrest, slippage, upregulated cyclin B1 and cdc2 activity, activation of SAPK/JNK cascade, disruption of tubular network;	Panzer et al., 2001
DU-145—human prostate carcinoma from brain metastasis, MCF-7—human breast adenocarcinoma, A549—human lung carcinoma, HepG2—human hepatocellular carcinoma, HT-29 human rectosigmoid adenocarcinoma	Chelidonine [1] and stylopine [33]	Chelidonine cytotoxic to DU-145, MCF-7, and HT-29 (IC_50_ 18.4, 8.30, 5.90 μM, respectively) whereas stylopine active against DU-145 and MCF-7 only (IC_50_ 13.9 and 16.6 μM, respectively);	Lee et al., 2007
Raji—human lymphoma	*C. majus* 40% ethanol (vodka) extract 10–200 μg/ml, equimolar mix of chelerythrine [9] and sanguinarine [12] 0.2–10 μg/ml	Complete cell kill by extract above 10 μg/ml (strongest among 61 tested traditional Russian and Siberian medicinal plant species) and by alkaloid mix above 5 μg/ml	Spiridonov et al., 2005
Murine Nk/Ly lymphoma	Chelidonine [1], chelerythrine [9], coptisine [31], sanguinarine [12] 1-40 μg/ml	Chelerythrine and sanguinarine strong cytotoxic (LD50 4, 25 and 6.2 μM, respectively), low activity of chelidonine and coptisine; strong correlation to DNA intercalation and genotoxicity (Comet assay)	Kaminskyy et al., 2006
Murine (L1210) and human (HL-60) leukemia	MeOH extract, 10–100 μg/ml	Cytotoxicity by apoptosis induction (DAPI staining), HL-60 cells more susceptible with 100% growth inhibition at 75 μg/ml (ca. 70% in L1210);	Nadova et al., 2008
Human T cell lymphoblastic leukemia	Chelidonine [1], sanguinarine [12] 1–10 μg/mL	Sanguinarine stronger cytotoxic (IC_50_ ca. 2.5 μM, 100% reached at 1.5 μM) than chelidonine (maximum 60% cell toxicity at a highest dose). Apoptosis induction comparable in two alkaloids (microscopy and cas-3 and Bax expression), but low genotoxicity of chelidonine; chelidonine but not sangunarine induced G2/M arrest;	Philchenkov et al., 2008
HL-60	Chelerythrine [9]	Cytotoxicity with IC_50_ of 2.6 μM, cell cycle arrest in G1, apoptosis induction (annexin, cas-8 activity) and necrosis;	Vrba et al., 2008
HepG2	Chelidonine [1] 0.1–100 μM	ca. 50% cell toxicity, dose independent; apoptosis and autophagy induction; decrease of telomerase activity and expression of hTERT subunit of telomerase (at 0.1 μM); long term treatment with 0.1 μM induced senescence.	Noureini and Wink, 2009
HeLa (human cervical cancer), PBMC (peripheral blood mononuclear cells)	Chelidonine [1] from ethanole extract isolated via MTT cytotoxicity guides chromatography, test conc. 22.5–37.5 μg/ml	Selectively cytotoxic to HeLa cells (LD_50_ ca. 85 μM); apoptosis induction (microscopy, annexin staining), cell cycle arrest (G_0_-G_1_), upregulation of p38, p53, cas-3, cas-9, Bax, APAF-1, down-regulation of PI3K, AKT, JAK3, STAT3, Bcl proteins, corroborating with RT-PCR;	Paul et al., 2013
HepG2, PBMC	Chelidonine [1], nano-encapsulated chelidonine [1]	HepG2 selective apoptosis promotion and cell cycle arrest (G_2_/M), modulation of apoptosis-associated genes transcription and protein levels; stronger effect of chelidonine in nanoformulation (e.g., IC_50_ in MTT assay was 2.91 vs. 5.45 μM), supported by its higher bioavailability in mice;	Paul et al., 2013
B16F10–murine melanoma, MCF-7-, 3T3—murine embryonal fibroblasts	Mixture of allocryptopine, chelidonine [1], protopine, sanguinarine [12], stylopine [33] (all 3.3 μg/ml)	Cytotoxic (up to 55%) to all lines, slightly selective to mouse melanoma; changes in alkaloid conc. in the medium and cell lysated was measured but no correlation of the cellular uptake to the toxicity was found;	Kulp et al., 2011; Kulp and Bragina, 2013
HeLa, HepG2, Caco2—human colon adenocarcinoma, human T cell leukemia—standard CCRF-CEM and multidrug resistant CCRF/ADR5000	MeOH extract from *Ch. herba* (HPLC tested) 5–4,000 μg/ml and pure chelidonine [1] 5–500 μM (concentrations of extract and chelidonine [1] didn't match in the experiments)	Cytotoxicity selective against some lines (most susceptible CCRF-CEM, moderate HepG2 and HeLa, resistant Caco2 and CCRF-ADR5000 to extract but not chelidonine) and apoptosis promotion; chelidonine much more effective than extract; enhanced doxorubicine toxicity in resistant cells, inhibition of ABC transporters; a number of cell death, efflux pumps, metabolism related genes were modulated—down regulation of MDR, upregulation of cell death and cell cycle control genes;	El-Readi et al., 2013
MCF-7	Chelidonine [1] 0.25–250 μM, berberine [28] 1–1,000 μM	Strong chelidonine cytotoxicity (LD_50_ ca. 8 vs. 54 μM berberine); apoptosis dominating at lower (<5μM) and autophagy at higher conc.; senescence induction at 0.05 μM for 33 days; telomerase suppression by chelidonine via down regulation of hTERT expression and inhibition of enzyme activity—interaction with G-DNA excluded;	Noureini et al., 2017
HepG2	Dichloromathane, ethanol, 50% ethanol, water extracts from *C. herba* (characterized by NMR and HPLC), diluted 1:501	Inhibition of cell proliferation, highest by ethanolic extract, followed by dichloromethane (no activity of water extract); activation of genes (whole genome microarray) related to drug metabolism, oxidative stress and cellular damage; no correlation to the alkaloid content;	Orland et al., 2014
PANC-1—human pancreatic cancer, PANC02—mouse pacreas cancer, HT-29—colon cancer, MDA-MB-231—human mammary gland cancer, BEAS-28—human bronchial eppithelium (non-cancerous), 3T3, PC-EM—primary endometrial cancer cultures (from surgery)	80% ethanol extract and *n*-hexane defatted ethanol extract 1–1,000 μg/ml (standardized by HPLC), Ukrain®	Cytotoxic to cancer lines (IC_50_ 19.4–57.8 μg/ml), with MDA-MB-231 significantly more resistant than other lines; lower cytotoxicity in 3T3 and PC-EM cells; pilot *in vivo* results of anti-metastatic effect against transplanted PANC02 in mice but no significant effect on tumor growth;	Capistrano et al., 2015
MDA-MB-231	Chelidonine [1] 0.3–10 μM	Inhibition of collagen-stimulated cell migration and invasion (but not fibronectin-induced); mechanisms include suppression of actin cytoskeleton reorganization, inhibition of integrin signaling via suppression of ILK association with other components of IPP complex essential for adhesion;	Kim et al., 2015
A431—human squamous cells epidermoid carcinoma	*C. majus* (parts used undefined) water extract, no standardization, 50–500 μg/ml	Increase of cas-3 activity, three-fold increase of apoptotic cells; modulation of cell death related genes—mRNA down regulation of Bcl-2, survivin and Mcl-1, upregulation of p21 and Bax; increase of p38, MEK, and ERK phosphorylation (activation), inhibition of NFκB activation;	Park et al., 2011
Human lung carcinoma (A549, H460), colon carcinoma (HCT116, SW480), breast (MCF-7, MDA-MB231), PBMC	*C. majus* whole plants, dried ethanol extract, standardized for alkaloids and polyphenols, 16–500 μg/ml	Selectively and differently toxic to cancerous cells, non-toxic to PBMC. IC_50_ between 44 and 143 μg/ml, depending on cell line, also synergistic activity with doxorubicin in its lower (1–2 μM) concentrations, antagonistic in higher dox doses; apoptosis induction (annexin, microscopy); G2/M arrest; inhibition of cell migration by scratch assay;	Deljanin et al., 2016
Jurkat E6.1 mutant—human T cell leukemia, MOLT4—human T cell lymphoblastic leukemia, U937—human histiocytic lymphoma, HEL92.1.7—human erythroleukemia, Raji—human Burkitt lymphoma, HL-60, A2780—human ovarian endometrioid adenocarcinoma, A549, human primary lung fibroblast (MRC-5 and WI-38), PBMC	Chelidonine [1], homochelidonine [1], 1–20 μM	Cytotoxic effect differing between cell lines (IC_50_ 1.8–5.0 μM), chelidonine stronger than homochelidonine, strongest effect toward MRC-5, and mutated Jurkat cells; induction of apoptosis with involvement of mitochondrial pathway; biphasic cell cycle arrest (G1, G2/M) by homochelidonine, but only G2/M by chelidonine; increase in H3 histone phosphorylation, activation of checkpoint kinases, inhibition of cell adhesion, interference with tubular skeleton, nuclei fragmentation;	Havelek et al., 2016a,b
SGC-7901 human gastric carcinoma	Chelidonine [1] 5–160 μM	Cytotoxic with IC_50_ at 23.13 μM; apoptosis inducing (microscopy); increase of histone H3 phosphorylation, time-dependent regulation of mitotic slippage associated protein levels—BubR1 (a checkpoint kinase), Cdk-1, cyclin B1; increase of cas-3 expression; G_2_/M arrest; microtubule polymerization inhibition causing mitotic catestrophe;	Qu et al., 2016
MCF-7	Berberine [28]-−50–500 μM, chelerythrine [9]-−2–25 μM, chelidonine [1]-−5–100 μM, papaverine, sanguinarine [12]-−2–25 μM	Different toxicity (IC_50_ = 2.5, 3.0, 8.0, 54.0, and 120.0 μM for chelerythrine, sanguinarine, chelidonie, berberine, and papaverine); strong inhibition of telomerase and hTERT expression by chelerythrine and sanguinarine, but only of hTERT expression by chelidonine; strong binding of chelerythrine and sanguinarine to telomeric G quadruplex, confirmed by docking;	Noureini et al., 2017
**REPORTS WHERE UKRAIN®WAS USED AS AN ACTIVE SUBSTANCE**			
Jurkat - various modifications (A3, J16, Cas-9 DN expressing, cas-8 and FADD-negative, CD95/TRAIL resistant A3, Bcl-2 overexpressing, cFLIP-L expressing)	Ukrain® (5–50 μg/ml) and alkaloid standards	Ukrain® disproved from being a derivative—just mixture of native alkaloids by mass spectrometry; strong apoptosis induction by Ukrain® and activation of caspases (3 and 8); apoptosis mediated by mitochondrial pathway; similar activity observed for chelidonine, chelerythrine, and sanguinarine;	Habermehl et al., 2006
Ewing sarcoma cell lines (CADO-ES-1, VH-64, STA-ET-1, STA-ET-2.1)	Ukrain® 0.05–50 μM, *C. majus* alcohol extract (commercial, standardized to 1.2 mg/g chelidonine [1])-−0.5–500 μM chelidonine [1]	Different cytostatic activity depending on the target line: STA-ET-1 and VH64 more sensitive (mean GI_50_ was 11.9 μM for Ukrain® and 12.3 μM for extract)	Lanvers-Kaminsky et al., 2006
human glioblastoma T60, T63	Ukrain®	Significant inhibition of cell proliferation at 10 μM, aopoptosis induction, increase of glial fibrillary acidic protein—crucial for lower malignancy adn tumor growth suppression;	Gagliano et al., 2006, 2007
Caki-1, ACHN—renal clear cell (Caki-1, ACHN) and papillary (Caki-2) renal metastatic carcinoma	Ukrain®	Reduced cell proliferation (10 μM), modulation of malignant phenotype and invasiveness, by inhibition of MMP activity and downregulation of secreted and upregulation of intracellular SPARC protein levels;	Gagliano et al., 2011
Pancreatic ductal adenocarcinoma (HPAF-II, HPAC, PL45)	Ukrain®	Reduced cell proliferation (10 μM), retaining of epithelial phenotype; decreased invasiveness by inhibition of MMP activity and downregulation of secreted and upregulation of intracellular SPARC protein levels; modification of mitotic spindle microtubules;	Funel et al., 2010; Gagliano et al., 2012
Murine (4T07, TUBO) or human (SKBR-3) breast cancer, 3T3	Ukrain®	Moderate (13–30%) cell death induction specific for cancerous lines. Inhibition of proliferation regain potential; induction of apoptosis (annexin, caspase-3 activity); verified by an *in vivo* experiment—diminished tumor growth in mice;	Bozeman et al., 2012
4T1—murine mammary gland cancer, B16F10	Ukrain®, in combination with several cytostatic drugs	IC_50_ was 40 μM (4T1) and 76 μM (B16F10), synergistic effect with bortezomid;	Savran et al., 2014

In more detail, quite many different, but mostly human cell lines were used as model systems (for references see Table 2), representing leukemias (Jurkat with several modifications to study certain cell death mechanisms), Raji, MT-4, MOLT-4, HL-60, U-937, HEL-92.1.7, CCRF/CEM, CCRF/ADR5000), colon carcinomas (Caco-2, HT-29, HCT116, SW480), breast cancer (MCF-7, MDA-MB231), pancreatic cancer (human PANC-1, murine PANC02), lung cancer ((A549, H460), prostate cancer (DU-145), cervical cancer (HeLa), ovarian carcinoma (A2780), liver cancer (HepG2), gastric cancer (SGC-7901), vulvar squamous cell carcinoma (A431), oesophageal squamous carcinoma (WHCO5), and mouse melanoma (B16F10). Non-cancerous lines, such as lung fibroblasts (MRC-5, WI-38), skin fibroblasts (Hs27) immortalized cells from mice (3T3), green monkey (Vero), humans (293N3S, HS-27, HaCaT), or SV-40 transformed bronchial epithelium (BEAS-2B) were also used.

From most of the published mechanistic studies a clear distinction can be established between mechanisms of action of chelidonine [1] and sanguinarine [12]/berberine [28]/chelerythrine [9].

Sanguinarine [12], chelerythrine [9], and berberine [28] possess strong affinity to binding G-quadruplex in telomeres which leads to blocking telomerase activity in fast proliferating cells (Noureini et al., 2017).

Unlike the quarternary alkaloids, chelidonine [1] is only a weak DNA intercalating agent and does not induce lethal mutations or DNA damage. Its mechanism of action is suggested to rely on interactions with spindle microtubules leading to cell cyle arrest and mitotic catastrophe, inhibition of ABC transporters thus abolishing multidrug resistance and finally modulation of gene transcription (telomerase, cell death-related, cell cycle-controlling). These properties combined with the stronger-acting intercalating alkaloids can make the whole alkaloid fraction a unique multifaceted agent targeting cancer cells.

For example, El-Readi et al. (2013) demonstrated complex interaction of chelidonine [1] and alkaloid-rich extract with several signaling pathways, including those responsible for cell cycle, cell death, and proliferation. Using microarrays confirmed by RT-PCR data, it was shown that a set of genes associated with multidrug resistance (e.g., ABC transporters and CYP) were significantly downregulated (from 50% in case of CYP3A4 to 99% in ABCG2). On the other hand, caspase-3 and 8 genes were upregulated (up to 27-fold). These results explain the strong augmenting of doxorubicin toxicity to resistant Caco-2 and CCRF/ADR5000 cells. Upon low-dose treatment with chelidonine [1] (20 μM), the LC_50_ decreased from 3.67 μM (Caco-2) and 32.16 μM (CCRF/ADR5000) to 0.42 and 7.4 μM, respectively. Similar results were obtained with an adequate concentration of extract (5 μg/ml) containing (in decreasing order) chelidonine [1], coptisine [31], stylopine [33], and protopine [37] as four major compounds and smaller amounts of other alkaloids. In the more resistant CCRF/ADR5000 cells the effect of extract was even stronger than pure chelidonine [1], suggesting that some of the other alkaloids are more efficient P-gp inhibitors. Induction of apoptosis was the major mechanism of cytotoxicity, also supported by increase of caspase activity and annexin staining.

These results support the potential of chelidonine [1] (and at least some other *C. majus* alkaloids) as ideal agents to overcome MDR as targeting both transport proteins, metabolic enzymes and pro-apoptotic pathways.

The interference of chelidonine [1] with the cell proliferation apparatus was also demonstrated using planarian stem cell model (Isolani et al., 2012) in which the gene expression of mcm2 (essential DNA replication factor) or inx-11 (a gap-junction protein essential for regeneration) was decreased by 20 μM of the alkaloid. The stem cells were affected but not differentiated populations such as neuronal or intestinal.

Quite a particular story pertains to the mentioned earlier patented product referred to as a semisynthetic derivative of alkaloids—a trimeric structure with thiophosphoric acid moiety connecting alkaloid molecules. It has been mentioned in the literature as NSC-631570 or Ukrain®. However, one of the major doubts regarding this product is its chemical identity (Panzer et al., 2000; Habermehl et al., 2006; Jesionek et al., 2016). Despite the earlier manufacturer claims, it couldn't be positively confirmed that it is indeed a thiophosphoric acid complex and at least in some batches, it appeared as a rich mixture of native alkaloids, that have been identified using reliable chromatographic and spectroscopic methods.

Several papers additionally report cytotoxic activity of Ukrain® on other cell lines such as prostate cancer (LNCaP, PC-3), glioblastoma (T60, T63, primary cancer line), pancreatic ductal adenocarcinoma (HPAF-II, HPAC, PL45, renal clear cell or papillary cell-derived lines (Caki-1, Caki-2, ACHN), melanoma (B16F10) and murine (TUBO, 4T1) or human (SKBR-3) breast cancer (see Table 2 for respective references).

However, due to the conflicting data about the chemical identity of this product, the previous results would have to be repeated or reinterpreted. If indeed Ukrain® is just a version of alkaloid mix, all the research done using it as an active substance would extend the scope of anticancer properties of *C. majus* native alkaloids. One of the early interesting findings is radiosensitizing influence of Ukrain® (Cordes et al., 2002, 2003) and it should be verified using non-modified alkaloid-rich extract and individual compounds to find out which of them or a combination would be responsible for this property. Also, other studies reporting Ukrain® as an active principle need re-evaluation with native alkaloids as probably constituting major component in the patented preparation. Moreover, Ukrain® remains the only preparation from *C. majus* that has a vast literature documenting its anti-cancer and also other properties in clinical (see the respective section in the present paper), pre-clinical and *in vivo* studies.

#### Anti-inflammatory and immunomodulating

Some of the versatile traditional uses of *C. majus* can be explained, as in many other herbs, by anti-inflammatory potential targeting various pathways in the organism as well as modulation of immune response. Both have been confirmed in many studies using *in vitro* cellular models, as well as *in vivo*.

The ability to inhibit inflammation or, in some cases, to stimulate immune response and mitigate excessive reactiveness can contribute to the postulated anticancer properties and improve symptoms of gastric disorders as well.

Schneider et al. (2016) used human colon cell line (NCM460) to check anti-inflammatory action of composite preparations containing *C. majus* marketed and popular in Europe and any of the individual components. *C. majus* extract was among the most potent in a couple of checked parameters, such as inhibition of IL-8, MCP, and I-TAC secretion, thus contributing significantly to the overall and apparently synergistic combination of active principles. This activity was likely to influence the clinical outcome mentioned below (Abdel-Aziz et al., 2017).

Mostly, the total extracts or isolated alkaloids were tested, but in our opinion, other components such as hydroxycinnamic derivatives, flavonoids and chelidonic acid are likely to contribute significantly. Chelidonic acid [45] was efficient in mouse models of ovoalbumin-elicited allergic rhinitis (Oh et al., 2011) and ulcerative colitis (Kim et al., 2012). This compound also attenuated inflammatory responses by reducing levels and gene expression of several mediators and enzymes in colon tissues (COX-2, HIF1α, PGE_2_) and in allergic mice (IL-4, IL-1β, COX-2, caspase-1, and increase of IFN-γ). In human mast cell line HMC-1 stimulated for inflammatory response by the phorbol ester (TPA) and calcium ionophore A23187, chelidonic acid [45] inhibited IL-6 expression by blocking NFκB (Shin et al., 2011).

Stylopine [33] added to the cell culture of in lipopolysaccharide-stimulated RAW264.7 macrophages was inhibiting production of several proinflammatory molecules such as nitric oxide, PGE_2_, TNF-α, IL-1β, and IL-6 (Jang et al., 2004). Also, iNOS and COX-2 protein levels were lowered. However, the cyclooxygenase activity inhibition was not selective. Chelidonine [1] and 8-hydroxydihydrosanguinarine [14a] in the same model inhibited NO production and iNOS and COX-2 gene transcription (Park et al., 2011). These results suggest the underlying inhibition of NFκB as potential mechanism but it wasn't investigated in this study. However, in HCT-1 colon cancer cell line treated with chelidonine [1], the NFκB activation was blocked by inhibition of IκBα degradation and nuclear translocation of p65 (an NFκB subunit) as well as mitogen-activated protein kinase pathway activation by blocking c-Jun N-terminal kinase and p38 phosphorylation (Zhang et al., 2018).

Alkaloid fraction and sanguinarine [12] were efficient against carrageenan-induced rat paw edema but chelerythrine [9] showed lower activity (Lanfeld et al., 1981). However, in the later study by Mikołajczak et al. (2015), various fractions of water extract at relatively high doses of 200 mg/kg body weight failed to alleviate the inflammation in the similar model. The crude water extract treatment actually aggravated the paw inflammation. Conversely, the extracts containing mainly coptisine [31] and chelidonine [1] were effective in hot plate test for antinociceptive properties that suggests a supramedullary way of action.

Chelerythrine [9] inhibited inflammatory and pain reaction in several *in vivo* and cell models employed by Niu et al. (2011). *In vivo*, i.p administration of the alkaloid (1–5 mg/kg) alleviated mouse ear edema, rat paw edema, and abdominal constriction (pain reaction). Also, the isolated peritoneal macrophages upon treatment with 0.0001–1 μg/ml chelerythrine [9] had dose dependently reduced PGE_2_ and COX-2 expression.

In NC/Nga mice model for atopic dermatitis induced by DNCB (1-chloro-2,4-dinitrobenzene), the hydroethanolic extract from aerial parts alleviated several measures of dermatitis such as itching behavior and skin severity (Yang et al., 2011). Interestingly, the oral administration at doses of 200 mg and 400 mg/kg were even more efficient than topical application as 1 and 2% smear. The IgE levels were put back to control values upon the higher dose oral administration and reduced by about 50% after topical treatment. Also, IL-4 and TNF-α serum levels were significantly reduced but remained higher than in control animals.

In an animal model of ovoalbumine-provoked asthma, chelidonine [1] suppressed eosinophile-mediated inflammation. The activity at the doses of 1 and 5 mg/kg body weight was similar to 0.5 mg/kg body weight dexamethasone. Among the several monitored parameters such as different pro-inflammatory cell population counts in bronchoalveolar lovage fluid and lungs, IgE, and cytokine protein and transcripts levels, some were inhibited even stronger than by dexamethasone (total BALF cells, Gr-1+/CD11b+ cells, IL-4), whereas others were inhibited either similarly or weaker than the standard drug. It suggests a specific mechanism involving STAT6 and Foxp3 transcription pathways (Kim et al., 2015). Yet another inflammation-based condition absent from list of traditional indications, in which *C. majus* was found to be efficient is arthritis (Lee et al., 2007). In the mice model of collagen-induced arthritis, aqueous-methanol extract at the oral doses of 40 and 400 mg/kg body weight, suppressed progress of joint damage as well as a set of studied inflammation-related cellular and biochemical parameters. In the higher dose regime, the incidence of arthritis decreased from 100 to <40% during 4 weeks. Cell invasion into lymph nodes, spleen, thymus and synovial fluid was inhibited in all organs, but most marked effect was in lymph nodes and joints. Among the tested T cells populations, there was an insignificant decrease of CD4+, CD8+, and CD3e+ cells, but a quite remarkable decrease of CD19+ B cells, almost to the level of non-arthritic animals. On the other hand, the number of regulatory CD4+CD25+ T cells increased significantly that suggests mobilizing adaptive response of the immune system to counteract and balance the excessive inflammatory processes. Suppression of inflammation mediators such as IL-6, TNFα, IFNγ was observed as well as lowered level of IgG and IgM but the latter only upon the higher dose treatment. However, the extract was not standardized an no particular compound or phytochemical class could be pinpointed as determining this potent anti-arthritic action.

A couple of reports indicate a potential of *C. majus* against Alzheimer's progression due to significant inhibition of acetylcholinesterase (AChE) without influence on butyrylcholinesterase (BuChE), which is a desired profile for potential drug-likeliness. The extract inhibited AChE *in vitro* by 98% at a concentration of 200 μg/ml and BuChE by only 13%. The isolated alkaloid 8-hydroxydihydrochelerythrine [10a] was the most active and AChE-selective (IC_50_ 0.61 μM and selectivity index 56.7) (Cho et al., 2006). In another study, chelidonine [1] and other active alkaloids were completely unselective with similar IC_50_ values against AChE and BuChE (Cahlíková et al., 2010). Interestingly, the specific AChE inhibiting activity by coptisine [31]-rich extract was discovered *in vivo* in a herbivore insect *Lymantria dispar* in which this activity contributes to killing the pest (Zou et al., 2017). By this property the plant is protecting itself from herbivore attack and it would be a feasible biological explanation of the therapeutic potential existence in the wild growing species.

The analgetic properties of alkaloids, mentioned before (Mikołajczak et al., 2015) can be explained by the observations of interaction with glycine transporters (Shin et al., 2003; Jursky and Baliova, 2011). The water extract inhibited the glycine-activated and enhanced glutamate-activated ion current in isolated rat periaqueductal gray (PAG) neurons studied by patch-clamp technique (Shin et al., 2003). Chelerythrine [9] and sanguinarine [12] selectively inhibited GlyT1 (but not GlyT2) in the micromolar concentrations (5–10 μM) while berberine [28] showed no inhibition in transfected HEK293T cells. GlyT1 inhibition was time-dependent, noncompetitive and increased with glycine concentration. Interestingly, chelerythrine [9] effect was reversible while sanguinarine [12] persisted through washing out (Jursky and Baliova, 2011).

### Clinical studies

Quite typically for many traditionally used medicinal plants, the clinical evidence of efficacy remains scarce and *C. majus* is no exception. Therefore, the relatively numerous pharmacological studies need rigorous verification by properly designed and supervised clinical studies. At present, most of the allegedly curative properties toward some important complaints remain unconfirmed, even if there is strong pre-clinical evidence. The millennia long tradition of use becomes confirmed in a significant part even if some mythical indications turned out to be invalid or perhaps have been misunderstood or distorted when passed through generations of practitioners and authors.

Several clinical studies exist on the compound preparation STW-5 containing 10% of *C. majus* herb extract (Drug-to-Extract Ratio = 1:3) as one of nine ingredients. The gastric disorders are the main indications and was evidenced both clinically and pharmacologically (Von Arnim et al., 2007; Abdel-Aziz et al., 2017). Certainly, there is no proof that *C. majus* was the essential ingredient in this preparation but the functional dyspepsia/postprandial distress syndrome it seems to be one of the most active (Abdel-Aziz et al., 2017) influencing acid regulation and antrum contraction as well as moderate mitigation of inflammatory reactions. The mechanisms behind these effects were also studied *in vitro* using cell lines showing anti-inflammatory activity (Schneider et al., 2016). IFN-γ dependent stat1 phosphorylation was postulated as a putative mechanism of action in which *C. majus* extract was among the averagely active, but apparently the whole composition had superior properties suggesting synergistic rather than additive effect.

A series of older clinical trials were performed on a product containing *C. majus* extract to test the efficacy in patients with bile tract and gall bladder complaints and gall stones. These studies, reviewed in detail in the EMA report, suggest significant improvement of many clinical parameters. These included: subjective complaints (pain attacks, feeling of fullness), physician's examination (sonography of gall bladder and liver, liver palpation, meteorism, jaundice) and laboratory tests (bilirubin, transaminases, blood sedimentation). The patient conditions in which the preparation containing *C. majus* alkaloids (in daily doses of ca. 0.2 mg alkaloid sum as chelidonine [1]) was administered with positive outcome were for example: cholelithiasis, cholangitis/cholecystitis, post-cholecystectomy, and alcohol toxic liver damage. In the entire area of gastrointestinal/hepatic complaints, one can estimate the number of human subjects involved in to date published clinical literature as exceeding 1,500.

An impressive number of studies suggest antitumor properties of the apparently semisynthetic product—Ukrain® that showed efficiency in several *in vitro* studies on an assortment of neoplastic and non-transformed cell lines (Capistrano et al., 2015) but it by large failed to demonstrate clinically relevant activity in humans. Several case reports have been published that suggest its beneficial action in a range of malignancies, such as melanoma, metastatic breast cancer, various carcinomas.

The randomized clinical trials (RCTs) using Ukrain® were reviewed over a decade ago (Ernst and Schmidt, 2005) with a conclusion that despite the intensive publishing activity of several groups testing Ukrain® against miscellaneous malignacies with very promising outcome, most of these data are full of shortcomings that prevent unequivocal credibility. Since then, only a few more studies have been realized but it is still far from definite resolving of this issue, especially in the situation that the chemical analyses, some of which were even co-authored by the inventor (Jesionek et al., 2016) proved that it is not the compound which was initially claimed.

Most of the RTCs published were full of inconsistencies or even appeared unreliable due to the insufficient information regarding the methodology. Despite the spectacular results in such malignancies as pancreatic or terminal colorectal cancer, serious doubts remain about the evidence provided in some of these trials. Some of the studies are so poorly documented that it would undermine the validity of the results. For example, missing or suspect randomization method, missing methods for tumor dimensions measuring, unclear protocols, subjective outcome evaluation, lack of proper statistics. Also, a great majority of the clinical trials and case reports were published in one journal. Nonetheless, these few reports on Ukrain® remain the only available clinical data focusing specifically on *C. majus* and strongly suggesting extraordinary antitumor potential.

Since the potential conflicts of interest, reliability of the clinical and chemical data of this product have been disputed repeatedly (Farrugia and Slevin, 2000, 2001; Nowicky, 2001; Ernst and Schmidt, 2005), more studies are indeed mandatory also in this aspect of *C. majus* as a source of highly potent substances with multidirectional mechanisms of action. Nonetheless, it should be borne in mind that approval of an extract/mixture based drug for clinical practice in such a sensitive field as oncology is highly improbable at the moment, and most likely it would remain so in the near future. Thus, further studies are mandatory that would focus on using individual alkaloids as lead structures for drugs that would target multiple anticancer mechanisms. On the other hand, clinical data on efficacy of various *C. majus* products used in complementary or adjuvant therapy may facilitate the tedious process of drug development without changing the existing paradigm of chemotherapy based on combination of chemically defined substances. Quite differently from the relatively safe (with limitations described below) gastrointestinal and cutaneous indications, the cautious policies of EMA and other agencies demonstrated in case of declared anticancer actions are reasonable and should be continued.

### Toxicology and safety issues

Repeatedly, studies and case reports occur that suggest hepatic injury/hepatotoxicity. It is especially important because one of the main indications of *C. majus* relates to liver and biliary tract disorders due to its cholagogue and hepatoprotective activities. The incidence of hepatotoxic cases and the possible clinical importance and safety issues have been reviewed recently (Pantano et al., 2017). Specifically, the results from animal studies are ambiguous and suggest a rather complex mixture of various mechanisms, such as inducing or alleviating oxidative stress or modulation of hepatic enzymes such as MAO and SOD, or slowing down mitochondrial respiration. The mitochondrial toxicity was related to DNA intercalating properties of sanguinarine [12] and chelerythrine [9].

In humans, numerous reports have been recorded since the 1990's. The main symptoms included cholestasis and mild to severe liver impairments with quite well documented causality in a majority of cases. In total, over 50 such cases have been reported from Europe, mostly from Germany (Etxenagusia et al., 2000; Stickel et al., 2003).

However, no certain constituent has been directly linked to the toxicity of the herb. On the contrary, it has been suggested that drug interactions rather than intrinsic toxicity are responsible for reported cases. Also, individual hypersensitivity or allergy has to be considered. Latex contained in the fresh plant is also possibly more toxic or allergenic than the dried material (EMA report, 2011).

In an *in vitro* study on HepG2 cells treated with various solvent extracts (Orland et al., 2014), the biotransformation and toxicity-related gene expression was enhanced, but the dichloromethane extract richest in chelidonine [1] and total alkaloids was the weakest inducer and least cytotoxic, whereas ethanolic extracts containing more coptisine [31] and sanguinarine [12] were more cytotoxic. However, the *in vivo* relevance of these results is uncertain. In rats, the high doses (up to 3g/kg body weight; Mazzanti et al., 2009) did not elicit any symptoms of hepatic injury and did not alter liver function.

Despite proven interaction of sanguinarine [12] and other alkaloids with DNA, no genotoxicity was observed using well established methods such as Ames tests or *in vivo* DNA damage (EMA report, 2011).

Nonetheless, uncontrolled internal use of unstandardized preparations should be discouraged and appropriate pharmacovigilance measures should be implemented to prevent unnecessary complications following *C. majus* administration. The comprehensive pharmacotoxicological investigation is also needed and should encompass establishing toxic doses range of various forms and preparations as well as individual constituents.

The European Medicine Agency therefore published the following recommendation:

“Two possible therapeutic indications were proposed for the monograph:

Traditional herbal medicinal product:

1. For symptomatic relief of digestive disorders such as dyspepsia and flatulence (oral intake)
2. For treatment of warts, callus and corns (cutaneous use)” (EMA, 2011).

However, based on the reported undesirable effects, including cases of liver damage, special precautions are necessary, especially during pregnancy and lactation, people suffering from liver diseases or taking liver-damaging drugs.

## Conclusions and future outlooks

Much effort has been put into the recognition of bioactive compounds contained in the extracts of *C. majus* and the mechanisms of their action. Numerous reports have been published about its effectiveness in treatment of different medical complaints, such as gastrointenstinal and hepatobiliary disorders or cutaneous ailments caused by a microbial or viral infection. Next to the huge number of scientific reports indicating the beneficial effect of *C. majus*, information about its potential toxic properties emerged. The use of herbal remedy carries the risk of adverse effects, which is why it is important to know the raw material. Official recommendations do not exclude the use of *C. majus* as traditional herbal medicinal product. Therefore, further research on the mechanisms of action during treatment should be carried out. It is also important to control the phytochemical composition of the raw material both in conventional growing conditions and *in vitro* cultures. The more so, because the potential risk of carcinogenesis and hepatotoxicity is still not well documented.

Finally, we conclude that the millenia long history of *Chelidonium* in folk, traditional, and official medicine is far from coming to the end. On the contrary, recent years witnessed a revival of advanced pharmacological and mechanistic approaches using both native complexes and individual components in discovery of the therapeutic potential of this herb. We are quite convinced that in the near future, at least some of the already known and evidence-based properties should and would find their place in officially recognized therapeutic procedures. Moreover, new discoveries should broaden the scope of traditional usage and deliver better preparations, especially combining different classes of active molecules such as proteins, alkaloids, chelidonic acid, and perhaps also polyphenols. To achieve it, much more joined and interdisciplinary efforts are necessary. Further, the plant's diversity on different levels must be thoroughly evaluated and optimal combination of the complex profile should be established for various target applications. As one of the oldest proverbs from Aristotle's book, also referring to *C. majus* original Greek name, “one swallow does not make a spring,” even if we observe a lot of “first swallows,” we urgently need more, to ensure rational exploitation of the huge potential hidden in the inconspicuous and common weed.

## Author contributions

SZ: Wrote phytochemical and bioactivity parts of the manuscript and critically reviewed and corrected the final manuscript; AJ-D: Wrote the ethno botanical and historical section of the manuscript; MW-K and IS: Contributed to the phytochemical and analytical parts of the manuscript; AJ: Contributed to the antimicrobial activity section of the manuscript; AM: Developed the entire concept of this review, wrote bioactivity and pharmacology sections, and critically read and corrected all parts of the paper.

### Conflict of interest statement

The authors declare that the research was conducted in the absence of any commercial or financial relationships that could be construed as a potential conflict of interest.

## Footnotes

^1^*The Plant List*. Available online at: http://www.theplantlist.org/1.1/browse/A/Papaveraceae/Chelidonium/ (Accessed Sept 29, 2017).

## Supplementary material

Supplementary Figure 1 Chelidonium majus has been highly valued by ancient physicians. Nowadays, it is still attracting researchers' and clinicians' attention for its unique composition of the yellow latex, rich in alkaloids and proteins.

Supplementary Table 1 The examples of conditions used to isolate alkaloids from *C. majus*.

Supplementary Table 2 The examples of application the separation techniques in analysis of alkaloids from *C. majus*.
