# Supplementary figures and images for: Greater Celandine's Ups and Downs−21 Centuries of Medicinal Uses of Chelidonium majus From the Viewpoint of Today's Pharmacology
Source: Front Pharmacol. 2018 Apr 11;9:299. doi: 10.3389/fphar.2018.00299 (PMC5912214; doi:10.3389/fphar.2018.00299)

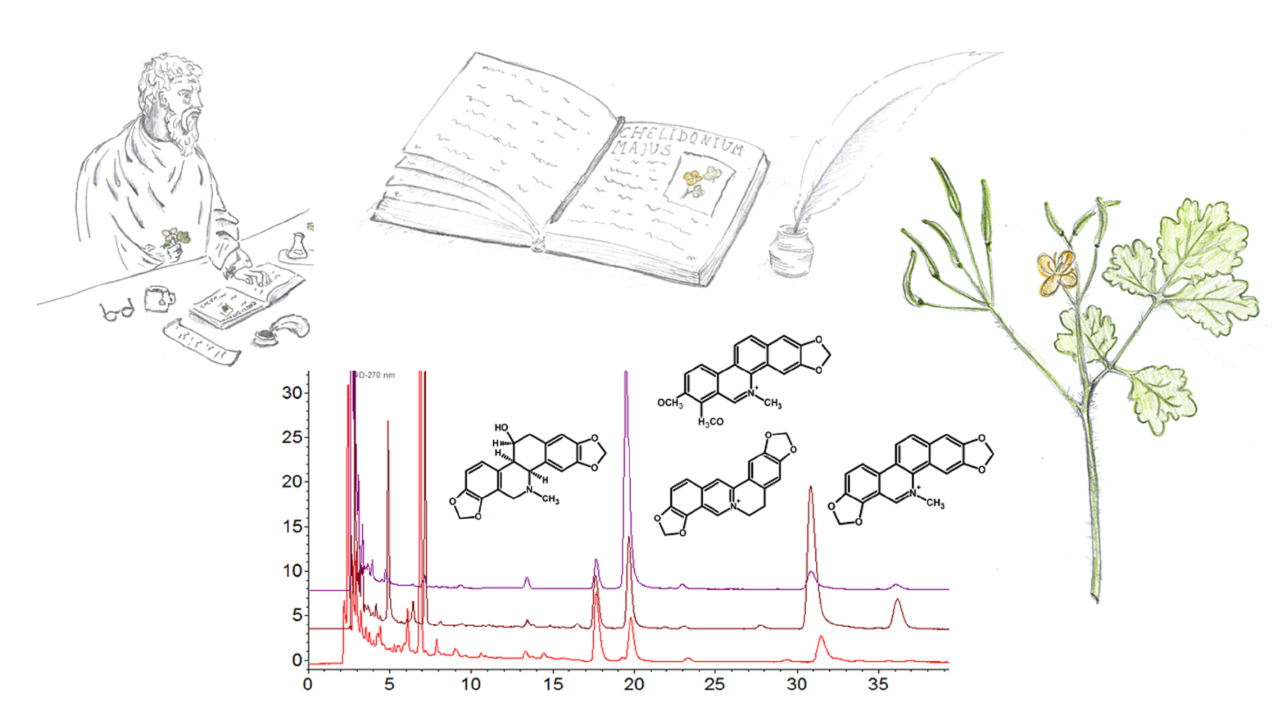

Supplement: Supplementary Figure 1 — Chelidonium majus has been highly valued by ancient physicians. Nowadays, it is still attracting researchers' and clinicians' attention for its unique composition of the yellow latex, rich in alkaloids and proteins. [file Image1.TIF]
