# Supplementary material for: Greater Celandine's Ups and Downs−21 Centuries of Medicinal Uses of Chelidonium majus From the Viewpoint of Today's Pharmacology
Source: Front Pharmacol. 2018 Apr 11;9:299. doi: 10.3389/fphar.2018.00299 (PMC5912214; doi:10.3389/fphar.2018.00299)
Supplement: Supplementary Table 1 — The examples of conditions used to isolate alkaloids from C. majus. [file Table1.DOCX]

Supplementary Table S1. The examples of conditions used to isolate alkaloids from *C. majus.*

| **Method** | **Conditions** | **References** |
| --- | --- | --- |
| SFE supercritical fluid extraction | supercritical CO_2_ and propylene glycol as co-solvent | Then et al., 2000 |
| Maceration 12 h and 30 min UAE ultrasound-assisted extraction | chloroform–ethanol (1:1) | Niu and He, 1991 |
| MAE microwaves-assisted extraction | water  40ºC, 25 min or 65ºC, 20 min | Then et al.,, 2000 |
| MAE | methanol–water–HCl (90:10:0.5) 60ºC, 5 min | Zhou et al., 2012 |
| SFE combined with ESE enhanced solvent extraction | First step: supercritical CO_2_  Second step: ethanol or isopropanol and diethylamine (9:1) | Gañán et al., 2016 |
| UAE | 0.05 M hydrochloric acid in methanol, 27ºC, 2 x10 min | Kursinszki et al., 2006 |
| Percolation | cold 1% acetic acid  adjusted to pH 6 with NaOH | Gołkiewicz i Gadzikowska, 1999 |
| UAE | methanol/diluted acetic acid at pH 3.5 (75:25), 30 min | Paulsen et al., 2015 |
| HRE heat reflux extraction | 12 M hydrochloric acid - 70% methanol aqueous solution (0.5:100) 60 min | Gu et al., 2010 |
| HRE | 12% acetic acid, 30 min | Seidler-Łożykowska et al., 2016 |
| water bath 30 min  UAE 20 min  HRE 30 min | 12 % acetic acid after extraction alkalized with 25 % ammonia to pH 8 further extracted with buthyl alcohol or dichloromethane or chloroform | Sárközi et al., 2006a  Sárközi et al., 2006b  Jesionek et al., 2016  Migas et al., 2012  Bogucka-Kocka and Zalewski, 2016 |
| Soxhlet | 70% ethanol | Bugatti et al., 1987  Colombo and Tome, 1991 |
| Soxhlet | methanol | Stuppner and Ganzera, 1995 |
| IL-ISFME ionic liquid - in-situ solvent formation microextraction | ion-pairing agent (KPF_6_) with a water-miscible ionic liquid 1-hexyl-3-methylimidazolium hexafluorophosphate (IL; [C_6_MIM][Br]) | Wu and Du, 2012 |
