# Supplementary material for: Greater Celandine's Ups and Downs−21 Centuries of Medicinal Uses of Chelidonium majus From the Viewpoint of Today's Pharmacology
Source: Front Pharmacol. 2018 Apr 11;9:299. doi: 10.3389/fphar.2018.00299 (PMC5912214; doi:10.3389/fphar.2018.00299)
Supplement: Supplementary Table 2 — The examples of application the separation techniques in analysis of alkaloids from C. majus. [file Table2.DOCX]

Supplementary Table S2. The examples of application the separation techniques in analysis of alkaloids from *C. majus*

| **Method** | **Sample/analytes** | **Stationary phase** | **Mobile phase** | **Detection** | **References** |
| --- | --- | --- | --- | --- | --- |
| 2D-HPTLC | herb, chelerythrine, protopine, berberine | CN F_254_ | 1) methanol- water (60:40) - 2% ammonia  2) diisopropyl ether - methanol (80:20) - 2% ammonia | DAD-densitometry | Petruczynik et al., 2007 |
| NP-TLC | leaf, stem, flower, root/coptisine, chelidonine, chelerythrine, sanguinarine, berberine | Si 60 F_254_ | methylene chloride-methanol (97:3); chloroform-methanol (60:30) | UV light at 254 and  365 nm, densitometry | Sárközi et al., 2006 |
| NP-TLC | aerial parts/coptisine, chelidonine, | Si 60 GF _254_ | 1-propanol–formic acid–water (90:1:9) | densitometry | Wagner et al., 1984  Then et al., 2000 |
| NP-TLC | aerial parts, roots/ sanguinarine, chelerythrine, berberine, coptisine, chelidonine, protopine | Si 60 F_254_ | two-step elution:  1) chloroform- methanol - water (70:30:4)  2) toluene-ethyl acetate - methanol (83:15:2) | UV light at 254 and  365 nm.  densitometry | Gadzikowska and Gołkiewicz, 1998;  Bogucka-Kocka and Zalewski, 2016 |
| NP-TLC in magnetic field | *C. majus* extracts/ allocryptopine, protopine, homochelidonine, chelidonine | Si 60 F_254_ | toluene-ethyl acetate-methanol (70:15:15) | densitometry | Malinowska et al., 2017 |
| NP-OPLC | *C. majus* extracts/ allocryptopine, protopine, chelidonine chelerythrine, chelilutine, sanguinarine, chelirubine | Si 60 F_254_ | Tertiary alkaloids, toluene–ethyl acetate-methanol, 70 + 15 + 15 (v/v) as mobile phase, quaternary alkaloids with toluene–ethyl acetate–methanol,  83 + 15 + 2 (v/v) | densitometry | Malinowska et al., 2005 |
| HPLC | roots/ sanguinarine, chelerythrine, berberine, | Silica (250 x 4.6  mm, 10 µm) | 0.005 M sodium acetate in methanol-1,4-dioxane-acetic acid (88:10:2), flow rate of 1.5 ml/min; | UV-Vis at 280 nm | Bugatti et al., 1987 |
| HPLC | *C. majus* plants/ corysamine, methoxychelidonine, allocryptopine, protopine, chelerythrine, berberine,  chelidonine, homochelidonine, oxysanguinarine, sanguinarine, dihydrochelerythrine, dihydrosanguinarine | Hypersil ODS column (100 x 4.6 mm, 5 µm) | water (adjusted to pH 7.5 with propylamine) –acetonitrile - methanol with 0.15 mM potassium iodide,  gradient elution from 50:20:30 to 15:55:30 in 15 min.  flow rate: from 0.8 to 1.5 ml/min in 15 min. | DAD | Han et al., 1991 |
| HPLC | *C.majus* tincture/ chelidonine, sanguinarine, chelerythrine, protopine | Discovery HS C18 (150 x 4.6 mm, 3 µm) | A: acetonitrile B: 0.030 mol/L formic acid in water,  gradient elution: from 0 to 5 min 15% A; from 5 to 20 min increase from 15% to 90% A; from 20 to 22 min 90% A, flow rate: 0.7 ml/min | DAD  MS/MS | Prosen Pendry, 2016 |
| HPLC | aerial parts, tincture/ chelidonine, sanguinarine, protopine, berberine, coptisine | Luna C18 (250 × 4.6 mm, 5 μm) | acetonitrile–methanol–30 mM ammonium formate, pH 2.80  (14.7:18:67.3); flow rate: 1 mL/min, temperature: 30ºC | DAD | Kursinszki et al., 2006 |
| HPLC | *C. majus* plant/ sanguinarine,  chelerythrine | RP 18 (250 × 4.6 mm, 5 μm) | A : 0.1% phosphoric acid  and 0.02% SDS (pH 3.5, adjusted by triethylamine); B: acetonitrile, gradient elution: 0–15 min,  30%–35% B; and 15–25 min, 35%-45% B; flow rate 1 mL/min,  temperature: 30ºC | Fluorescence: excitation 330 nm, emission 555 nm. | Wu and Du, 2012 |
| HPLC | leaves/ chelidonine,  berberine, sanguinarine, chelerythrine, coptisine, | Nucleosil RP-18 (5 µm) | A: acetonitrile; B: 10 mM (NH_4_)_2_SO_3_ with 0.2% triethylamine adjusted with acetic acid to pH 4.0; C: methanol,  gradient elution: 0 min: 5% A, 90% B, 5% C; 24 min: 70% A, 10% B,20% C; flow rate: 1.0 mL/min. | DAD  MS | Paulsen et al., 2015 |
| HPLC | herb/ chelidonine coptisine sanguinarine berberine chelerythrine | ZORBAX Poroshell 120 SB-C18 (3×100 mm, 2.7 μm) | A: 30 mM ammonium formate (pH 2.8) , B: acetonitrile:methanol 14.7:18.0;  gradient elution: from 20% to 60% B in 16 min; flow rate 0.5 mL/min,  temperature: 40ºC | DAD | Seidler-Łożykowska et al., 2016 |
| HPLC | aerial parts, flowers, fruits, leaves, roots, stems/ protopine, chelidonine, coptisine, stylopine, sanguinarine, berberine, chelerythrine | Luna C18 (250 × 4.6 mm, 5 μm) | acetonitrile-methanol-30 mM ammonium formate (pH 2.8)150:180:670; flow rate 0.8 mL/min. | UV/vis detector | Borghini et al., 2015 |
| HPLC | aerial parts, terrestrial parts/ chelidonine, cheleritrine, sanguinarine, berberine | RP 18 (250 × 4.6 mm, 5 μm) | A: heptanesulfonic acid (0.01 M) and triethylamine (0.1 M) in water acidified with formic acid (pH 2.5) B: acetonitrile;  gradient elution: 0 min: 75% A; 1 min: 68% A; 2 min: 57.5% A; 4.5 min: 40% A; 12 - 40 min: 20% A;  flow rate 1 mL/min | DAD | Gañán et al., 2016 |
| HPLC | aerial parts/ dihydroberberine, protopine, allocryptopine, chelidonine, coptisine, tetrahydrocoptisine, tetrahydroberberine, berberine, norchelidonine, chelerythrine | Luna C18 (150 x 1.0 mm, 3 µm) | A: 1% acetic acid in water, B: methanol  gradient elution: 20% to 50% B during 30 min, 80% B at 40 min. flow rate 20 µL/min | DAD–ESI/MS^n^ | Grosso et al., 2014 |
| CE | aerial parts/ sanguinarine,  coptisine,  chelerythrine,  stylopine ,  chelidonine,  protopine, allocryptopine | polyimide-coated fused silica capillary, 50 cm x 75 µm | 20 mM sodium phosphate pH 3.1 | ultraviolet light-emitting diode-induced native fluorescence: excitation 280 nm, emission 200 to 600 nm. ESI-MS | Kulp et al., 2011 |
| CE | *C. majus* plant/ sanguinarine, coptisine, chelerythrine, berberine, chelidonine,  protopine,  allocryptopine,  stylopine | fused-silica capillary, 35 cm x 50 µm | 500 mM Tris–H_3_PO_4_ buffer (pH 2.5) with 50% methanol and 2mM HP-β-cyclodextrin. | DAD | Zhou et al., 2012 |
| CE | aerial parts/ coptisine,  berberine, protopine, chelidonina,  stylopine | fused-silica capillary, 70 cm × 50 µm | citric acid-Na_2_HPO_4_ buffer (pH 5.5) and β-cyclodextrin (12.5 mM) | UV/vis detector | Stuppner and Ganzera, 1995 |

*OPLC optimum performance laminar chromatography
